# EIF4A3-induced circUBAC2 promotes lung cancer progression via regulation of the Hippo signaling pathway

**DOI:** 10.1186/s11658-026-00912-0

**Published:** 2026-04-05

**Authors:** Fan Meng, Xiaokang Zhang, Xinlin Wang, Binqiang Qiu, Dingcheng Zeng, Xiao Huang, Jianping Liu, Haiwu Wu, Kaiwang Cui, Bin Zhong, Weiyou Liu, Xiangwen Gong, Xin He

**Affiliations:** 1 Jiangxi Provincial Branch of China Clinical Medical Research Center for Geriatric Diseases, The First Affiliated Hospital of Gannan Medical University, Ganzhou, Jangxi Province People’s Republic of China; 2 The First Clinical Medical College of Gannan Medical University, Ganzhou, People’s Republic of China; 3 Department of Respiratory and Critical Care Medicine, Ganzhou Key Laboratory of Respiratory Diseases, Ganzhou Institute of Respiratory Diseases, The Fifth People’s Hospital of Ganzhou, Ganzhou, Jiangxi China; 4 Department of Respiratory and Critical Care Medicine, The First Affiliated Hospital of Gannan Medical University, Ganzhou, People’s Republic of China

**Keywords:** circUBAC2, EIF4A3, OTUB1–YAP axis, 14-3-3 proteins, Lung adenocarcinoma

## Abstract

**Background:**

Circular RNAs (circRNAs) are stable noncoding RNAs involved in cancer, yet their mechanisms in lung adenocarcinoma (LUAD) remain unclear. This study explores the oncogenic role of circUBAC2 in LUAD progression.

**Methods:**

Differentially expressed circRNAs were screened by microarray. circUBAC2 expression/location was analyzed via quantitative real-time PCR (qRT-PCR), RNA in situ hybridization (RNA-ISH), and fluorescence in situ hybridization. Functional impacts were evaluated through wound healing, Transwell, EdU, colony formation, and xenograft models. circUBAC2 targets were identified by RNA-sequencing/proteomics, with protein interactions assessed via western blotting, immunoprecipitation, and ubiquitination assays. Molecular interactions were examined using dual luciferase, chromatin Immunoprecipitation (ChIP), and RNA pull-down.

**Results:**

circUBAC2 was upregulated in LUAD tissues and correlated with poor prognosis. It promoted LUAD cell proliferation, migration, and invasion in vitro, as well as tumor growth/metastasis in vivo. Mechanistically, circUBAC2 competitively bound 14-3-3 to release YAP for nuclear translocation while scaffolding OTUB1–YAP interactions to enhance YAP deubiquitination and stabilization, collectively activating TEAD-mediated oncogenic transcription. EIF4A3 facilitated circUBAC2 biogenesis by binding flanking intronic regions.

**Conclusions:**

circUBAC2 drives LUAD progression via YAP signaling, positioning it as a therapeutic target and prognostic biomarker.

**Supplementary Information:**

The online version contains supplementary material available at 10.1186/s11658-026-00912-0.

## Background

The incidence and mortality of lung cancer rank first among all tumors [1]. The high incidence and high mortality of lung cancer have had a serious impact on global health [2]. Traditional treatments are no longer able to significantly prolong the survival of patients with lung cancer. Surgical treatment only plays a role in patients with nonadvanced lung cancer [3]. However, most patients with lung cancer are already in the late stage of the disease when diagnosed [4], precluding their surgical treatment. For patients with advanced lung cancer, chemotherapy remains an option. However, this treatment results in extremely limited improvement in the survival time and quality of life of patients with lung cancer [5]. The emergence of immunotherapy and targeted therapy heralded the beginning of the precision therapy era in lung cancer treatment. These treatments provide new hope and choices for patients with lung cancer. Nevertheless, the rapid development of drug resistance in patients with lung cancer remains a major challenge [6]. Therefore, rigorous research into the molecular basis of lung cancer is required. The success of lung cancer treatment correlates highly with its pathological type and staging. Habitually, lung cancer is classified as small cell lung cancer (SCLC) or non-small cell lung cancer (NSCLC) in clinical practice, accounting for approximately 15% and 85% of cases, respectively [7]. As a pathological subtype of NSCLC, lung adenocarcinoma is characterized by a high rate of mutation of the EGFR gene (encoding epidermal growth factor receptor). Despite the development of numerous drugs targeting EGFR, drug resistance still poses a serious challenge. Moreover, only a small fraction of patients with lung adenocarcinoma benefit from such drugs [8]. Therefore, it is still necessary to search for new targets to treat lung adenocarcinoma.

Circular RNA (circRNA) is a special type of RNA. Unlike traditional linear RNA, circRNA molecules have a closed circular structure that is not affected by RNA exonucleases, resulting in more stable expression and less degradation [9]. Most circRNAs do not have a protein coding function. Many circRNAs exert their biological effects by regulating target gene expression by sponging microRNAs (miRNAs) that inhibit translation of the target mRNA. Previously, we showed that hsa_circ_0021727 (circ-CD44) promoted the progression of esophageal squamous cell carcinoma (ESCC) by sponging miR-23b-5p, thereby activating the TGF-beta activated kinase 1 (MAP3K7) binding protein 1(TAB1)/nuclear factor kappa B (NFκB) pathway [10]. circRNAs also interact with proteins. For example, circNDUFB2 modulates cellular immune response and protein ubiquitination and degradation, thus affecting insulin like growth factor 2 mRNA binding protein (IGF2BP) degradation and antitumor immunity activation during the progression of NSCLC [11]. Research has shown that a small fraction of circRNAs also encode proteins. circAKT3 encodes AKT3-174aa, which interacts with phosphorylated pyruvate dehydrogenase kinase 1 (p-PDK1) as a molecular decoy [12]. Despite being important molecules in the field of cancer, circRNAs still have many unclear mechanisms that require exploration.

The DEAD-box protein, eukaryotic translation initiation factor 4A3(EIF4A3), releases sub-structures from the 5′-untranslated region of mRNAs, thereby catalyzing translation [13]. Evidence suggests that EIF4A3 can regulate the expression of circRNAs. Mediated by E2F1 and EIF4A3, circRNA circSEPT9 promotes the carcinogenesis and development of triple-negative breast cancer [14]. Jiang et al. revealed that EIF4A3-mediated circARHGAP29 binds directly to IGF2BP2, which contributes to *LDHA* (encoding lactate dehydrogenase A) mRNA stability; LDHA plays an important role in tumor cell apoptosis and autophagy [15, 16]. However, the molecular mechanism by which EIF4A3 regulates circUBAC2 expression is currently unclear.

In this study, circUBAC2 was upregulated in lung adenocarcinoma tissues and negatively correlated with patient prognosis. Its expression was regulated by EIF4A3. Functionally, circUBAC2 promoted the migration, invasion, and proliferation of lung adenocarcinoma cells. Mechanistically, circUBAC2 facilitated YAP nuclear translocation by binding to tyrosine 3-monooxygenase/tryptophan 5-monooxygenase activation protein gamma (14-3-3-γ). In addition, circUBAC2 enhanced the interaction between OTU structural domain ubiquitin aldehyde binding 1 (OTUB1) and YAP, enabling OTUB1 to deubiquitinate YAP and stabilize it. These findings demonstrate that circUBAC2 plays a crucial role in lung adenocarcinoma progression.

## Methods and materials

### Samples of clinical tissues

In total, 20 lung adenocarcinoma tissue specimens were acquired from the First Affiliated Hospital of Gannan Medical University from patients that required surgery, but had not been treated using radiation therapy or chemotherapy. The samples were collected between January 2019 and December 2019. The dissected tissue samples were placed in a −80 ℃ freezer immediately after surgery. The process of obtaining surgical specimens received approval from the Ethics Committee of the First Affiliated Hospital of Gannan Medical University, and all participants guaranteed their consent in writing.

The tissue microarray, comprising 75 paired lung adenocarcinoma samples and their adjacent noncancerous tissues, was acquired from Shanghai Outdo Biotech Co., Ltd. (Shanghai, China).

### circRNA microarray

Three pairs of tissue samples were used for circRNA microarray hybridization and analysis of the acquired data. First, we extracted total RNA from the paired specimens. Second, we removed linear RNA to enrich the proportion of circRNA via RNase R digestion (Epicenter, Madison, WI, USA). Then, we amplified the obtained circRNA utilizing the random start method (Arraystar Super RNA Labeling Kit; Arraystar, Rockville, MD, USA), followed by transcription into fluorescent cRNA. Finally, an Arraystar human circRNA array (8 × 15 K) was used for hybridization of the labeled cRNA. The array slide was rinsed and then scanned employing an Agilent G2505C scanner (Santa Clara, CA, USA). Agilent feature extraction software (version 11.0.1.1) performed data analysis of the resultant images.

### RNA in situ hybridization (RNA-ISH) of circRNAs

ISH was carried out employing standard protocols [17]. The tissue microarray was subjected to deparaffinization, rehydration via gradient ethanol concentrations, and digestion using proteinase K (20 μg mL^−1^; Roche Diagnostics, Indianapolis, IN, USA). The array was then subjected to formaldehyde fixation (Thermo Scientific, Rockford, IL, USA), washed two times using 0.13 M 1-methylimidazole, and then fixed for a second time using 1-ethyl-3-(3-dimethylaminopropyl) carbodiimide (EDC; Thermo Scientific). Endogenous peroxidase in the array tissue was then blocked using H_2_O_2_, followed by treatment with prehybridization buffer. The slides were then subjected to hybridization with 200 nM locked nucleic acid (LNA)-modified digoxigenin (DIG).

### Immunohistochemical staining and scoring

Paraffin-embedded tissue was subjected to dewaxing and hydration, followed by retrieval of antigens by heating in citrate buffer (10 mM citric acid, pH 6.0). Then, 5% animal serum was used to block the tissue, which was then reacted with primary antibodies (1:1500 dilution) at 4 °C overnight. The color was developed using 3,3′-diaminobenzidine (DAB). Hematoxylin was then used to counterstain the nuclei. Images of the tissue slides were captured under a microscope (Zeiss, Oberkochen, Germany).

Staining of the microarray and its immunoreactivity were scored independently by two experienced pathologists who were not informed concerning patient outcomes or clinical data, utilizing the listed criteria below. The intensity of immunostaining was scored as 0 (none), 1 (weak), 2 (moderate), and 3 (strong). The proportion of cells showing immunoreactivity was recorded as 0 (none), 1(< 20%), 2 (20–50%), 3 (51–75%), and 4 (> 75%). The degree × intensity staining rank was calculated and utilized to ascertain the cutoff values for the low and high expression groups. A final score < 6 identified the low expression group, and a score ≥ 6 identified the high expression group.

### Culture of cells

This study used the human lung adenocarcinoma cell lines A549 and H1975 (American Type Culture Collection, Manassas, VA, USA). The cells were incubated at 37° C and 5% CO_2_ in a humidified environment. Roswell Park Memorial Institute (RPMI) 1640 medium (Gibco, Grand Island, NY, USA) with 10% fetal bovine serum (FBS; Gibco) was used to grow the cells.

### Quantitative real-time reverse transcription PCR (qRT-PCR)

Tissue and cell total RNA was extracted employing RNAiso Plus (Takara, Shiga, Japan). Reverse transcription of the RNA to cDNA employed PrimeScript RT Master Mix (Takara, Dalian, China). RNA levels were determined using qPCR employing the SsoFast EvaGreen Supermix (Bio-Rad Laboratories, Hercules, CA, USA) or TB Green Premix Ex Taq II (Takara) with reaction conditions comprising 95 °C for 30 s, 40 cycles of 95 °C for 5 s and 60 °C for 30 s, plus a final dissociation step. The detected internal reference gene was *GAPDH* (encoding glyceraldehyde-3-phosphate dehydrogenase). Table S2 lists the primer sequences.

### Cell transfection

Synbio Technologies (Suzhou, China) designed and synthesized the circUBAC2 overexpression vector, the lentiviral vectors encoding short hairpin RNA (shRNA), and their associated negative controls. Shanghai Scigrace Biotech (GenePharma, Shanghai, China) synthesized the plasmids encoding short interfering RNAs (siRNAs) and the *EIF4A3* overexpression plasmid. Cells were transfected with the above vectors with the aid of Lipofectamine 3000 (Invitrogen, Carlsbad, CA, USA) following the supplier’s guidelines. Table S3A lists the siRNA and shRNA sequences.

### RNA FISH immunofluorescence microscopy.

Lung adenocarcinoma cells were fixed and permeabilized, followed by hybridization overnight in the dark at 37 °C with Cy-3-conjugated circUBAC2 probes (RiboBio, Guangzhou, China). The cells were then rinsed in saline-sodium citrate (SSC) buffer at 42 °C. Blocking buffer (phosphate-buffered saline Tween 20 [PBST] containing 5% bovine serum albumin) was incubated with the samples for 30 min at room temperature. Next, the cells were cultured in the presence of primary antibodies for 60 min at room temperature, and then reacted with Alexa Fluor 594- or 488-labeled secondary antibodies and 4′,6-diamidino-2-phenylindole (DAPI) (Vector Laboratories) for half an hour. A confocal microscope was used to acquire images of the cells. Table S4 lists the antibodies used.

### Assessment of cell proliferation

The 3-[4,5-dimethylthiazol-2-yl]-2,5-diphenyltetrazolium bromide (MTT) assay was utilized to investigate the proliferation of lung adenocarcinoma cell. Cells transfected with various constructs were added to the wells of a 96-well plate with 20 µL of MTT solution (5 mg mL^−1^; MTT Cell Proliferation and Cytotoxicity Assay Kit, BOSTER, Wuhan, China). After incubation for 4 h, we added 100 µL of dimethyl sulfoxide. A microplate reader determined the optical density at 490 nm.

### Assessment of colony formation

Transfected cells (1000 cells per well) were added to the wells of six-well plates and grown for 14 days. The cells were then subjected to 75% ethanol fixation and 0.2% crystal violet staining. Finally, microscopically, the stained colonies were enumerated to determine the colony formation rate.

### Assessment of 5-ethynyl-2′-deoxyuridine (EdU) immunofluorescence

Following the supplier’s guidelines, the Edu assay was carried out using a Cell-Light EdU DNA Cell Proliferation Kit (RiboBio, Guangzhou, China). The EdU reagent was incubated with transfected lung adenocarcinoma cells for 180 min. Following fixation and permeabilization, cell staining was performed using DAPI and anti-EdU reagents. Finally, we acquired images under a fluorescence microscope.

### Assessment of cell invasion

The Transwell chamber membrane was precoated using Matrigel (100 μL; BD Bioscience, San Jose, CA, USA), followed by medium addition to the lower chamber. The transfected cells were added with serum-free medium (100 μL). The upper chamber received these cells, and the lower chamber received complete medium. The loaded chambers were incubated at for 1 day in 5% CO_2_ at 37 °C. Finally, the invasive cells (those that had moved across the membrane) were subjected to 4% paraformaldehyde fixation and 0.1% crystal violet solution staining. An oil immersion inverted fluorescence microscope was employed to image and count the cells.

### Assessment of three-dimensional (3D) spheroid formation

Transfected cells were grown in ultra-low attachment (ULA) round-bottom 24-well plates for 4 days, during which time they could form tumor spheroids. After 4 days, each well received basement membrane matrix (BMM, 100 µL; Corning Inc. Corning, NY, USA), which was allowed to solidify for 60 min at 37 °C. Finally, each well received 100 µL of 10% FBS-containing medium, followed by incubation in 5% CO_2_ at 37 °C. Images of the spheroids were capture using an inverted microscope.

### Assessment of wound healing

Transfected cells were grown in serum-free medium in a six-well plate. When a cell monolayer formed, a sterile pipette tip was employed to score a linear wound across the cell monolayer. Photographs of the wounds were taken using a microscope at 0 h and 1 day post-wounding. The change in the width of the wound between the two time points was used to ascertain the extent of cell migration.

### Western blotting analysis

Radioimmunoprecipitation assay (RIPA) buffer was used to lyse the transfected cells, which were then centrifuged and the supernatant retained. The supernatant was added with 1% protease inhibitors (ComWin Biotech, Beijing, China). A bicinchoninic acid (BCA) Kit (Beyotime Biotechnology, Haimen, China) was employed to ascertain the lysate protein content. SDS–PAGE (10% or 8%) was used to separate equal amounts of proteins, which were then electrotransferred onto 0.45 µm polyvinylidene fluoride (PVDF) membranes (Roche, Indianapolis, IN, USA). Nonspecific binding to the PVDF membranes was carried out for 60 min using 5% nonfat milk. Primary antibodies were then added to the membranes, followed by overnight incubation at 4 °C. The membranes were then rinsed at least thrice with Tris-buffered saline–Tween 20 (TBST; 10 min for each rinse). Goat anti-rabbit or goat anti-mouse secondary antibodies were then added to the membranes and incubation was continued for 60 min. After three further TBST rinses, SuperSignal West Femto Agent (Millipore) was employed for immunoreactive protein band detection, followed by visualization employing the Chemical Mp Imaging System (Bio-Rad). Table S4 lists the antibodies utilized in this assay.

### RNA pull-down assay

First, 1 × 10^7^ cells were rinsed using chilled PBS, followed by lysis using 500 μL of co-immunoprecipitation (co-IP) buffer (Thermo Scientific) containing an RNase inhibitor, phosphatase inhibitors, and proteinase inhibitors (Invitrogen). Then, to the lysate was added 3 μg of biotinylated DNA oligo probes designed against the circUBAC2 backsplice junction sequence (sense) or their complementary probes (antisense) and incubated at room temperature for 120 min. Next, 50 μL of rinsed streptavidin C1 magnetic beads (Invitrogen) was added to each reaction and incubated for 60 min at room temperature. The beads were rinsed with co-IP buffer five times. Ultimately, the retrieved proteins were subjected western blotting or mass spectrometry analysis.

### Mass spectrometry (MS) analysis

For MS analysis, protein samples obtained from RNA pull-down were firstly resolved employing SDS–PAGE and stained using Coomassie brilliant blue. We then excised the gel strips and sent them to PTMBIO company (Hangzhou, China) for protein identification by MS analysis.

### RNA immunoprecipitation (RIP)

A Magna RIP RNA-Binding Protein Immunoprecipitation Kit (Millipore, Billerica, MA, USA) was utilized to carry out RIP following the supplier’s guidelines. qRT-PCR was then employed to quantify the co-precipitated RNA.

### Co-immunoprecipitation (co-IP) assay

An IP kit (Thermo Fisher Scientific, San Diego, CA, USA) was utilized to immunoprecipitate proteins to investigate the binding among circUBAC2, YAP, and 14-3-3-γ, followed by western blotting detection.

### Chromatin immunoprecipitation (ChIP)

ChIP was performed utilizing a SimpleChIP Plus Sonication Chromatin IP Kit (CST, Danvers, MA, USA) subject to the vendor's guidelines. Crosslinking of A549 cells was carried out using 1% formaldehyde for 10 min, followed by glycine quenching. Sonication was used to shear the DNA into 200–500 bp fragments. Anti-EIF4A3 and IgG antibodies were used to carry out immunoprecipitation from the nuclear extract. Following purification of the DNA fragments, qRT-PCR was carried out employing specific primers.

### Luciferase reporter assay

TEAD activation was determined utilizing a (TEAD) luciferase reporter (Addgene, Watertown, MA, USA), and a TK–Renilla plasmid was used for normalization of the firefly values (Promega, Madison, WI, USA). Both were transfected into A549 and H1972 cells with the aid of Lipofectamine 3000 (Invitrogen, Waltham, MA, USA) following the supplier’s guidelines. Cells were treated for 24 h with DMSO, 10 nM 2,3,7,8-tetrachlorodibenzodioxin (TCDD) (Sigma-Aldrich, St. Louis, MO, USA), and the indicated inhibitors. Renilla and luciferase signals were determined at 24 h after transfection employing a Dual Luciferase Reporter Assay Kit (Promega).

### RNA sequencing (RNA-seq)

ESCC cell total RNA extraction utilized the TRIzol^®^ reagent (Invitrogen, Shanghai, China) following the supplier's guidelines. A TruSeq Stranded Total RNA with Ribo-Zero Gold kit (Illumina, San Diego, CA, USA) was employed to construct the sequencing libraries following the supplier's guidelines. The Illumina sequencing platform HiSeq™ 2500 (Aksomics, Shanghai, China) was then used to sequence the libraries.

### Cycloheximide (CHX)-chase assay

Protein half-life was determined via CHX-chase assay. Briefly, cells co-tansfected with circUBAC2 overexpression plasmid and OTUB1 siRNA(48 h post-infection) were treated with 100 μg mL^−1^ cycloheximide (S7418, Selleck). Cells were lysed at indicated timepoints (0–8 h) in RIPA buffer containing protease inhibitors. Lysates were analyzed by western blot using anti-YAP and anti-GAPDH antibodies. Band intensities were quantified using ImageJ.

### Ubiquitination assay

Cells stably transfected with indicated plasmids (vector control, circUBAC2 overexpression, or circUBAC2 + OTUB1 siRNA) were pretreated with 20 μM MG-132 (S1748, Beyotime Biotechnology, China) for 8 h prior to lysis to prevent proteasomal degradation. Protein ubiquitination was analyzed by immunoprecipitation using a YAP-specific antibody, followed by immunoblotting with an anti-ubiquitin antibody to assess both exogenous and endogenous ubiquitination levels.

### Animal studies

Guangdong Medical Laboratory Animal Center (MLAC) provided male BALB/c nude mice (20 ± 2 g). All animal experiments were carried out following the principles and procedures in the Gannan Medical University Guide for the Care and Use of Animals. The First Affiliated Hospital of Gannan Medical University Animal Ethics Committee provided approval.

Subcutaneous injection of transfected cells was made into the left side of the axilla to observe the growth of the tumor, which was assessed by determining the xenograft volumes once per week, calculated as: (volume) = 1/2 × (long axis) × (short axis).

Establishment of a lung metastasis model was accomplished by tail vein injection of stable firefly luciferase-expressing cancer cells into BALB/c nude mice (4 weeks old). Six weeks later, an in vivo imaging system was utilized to obtain images of bioluminescent lung tumor metastases. Following humane sacrifice, mouse lung tissue was dissected and the number of lung metastases was determined; the tissue was also made into histological sections, followed by hematoxylin and eosin (HE) staining.

### Statistics

Data were analyzed statistically using GraphPad Prism 9.1 (GraphPad Software Inc., La Jolla, CA, USA) together with SPSS (version 26.0, IBM Corp., Armonk, NY, USA). Student’s *t*-test and/or a Chi-squared test were employed to analyze the data. Data are presented as the mean ± SD. A difference with *P* < 0.05 indicated statistical significance.

## Results

### Characterization of circUBAC2 in lung adenocarcinoma cells and tissues

**Fig. 1:**
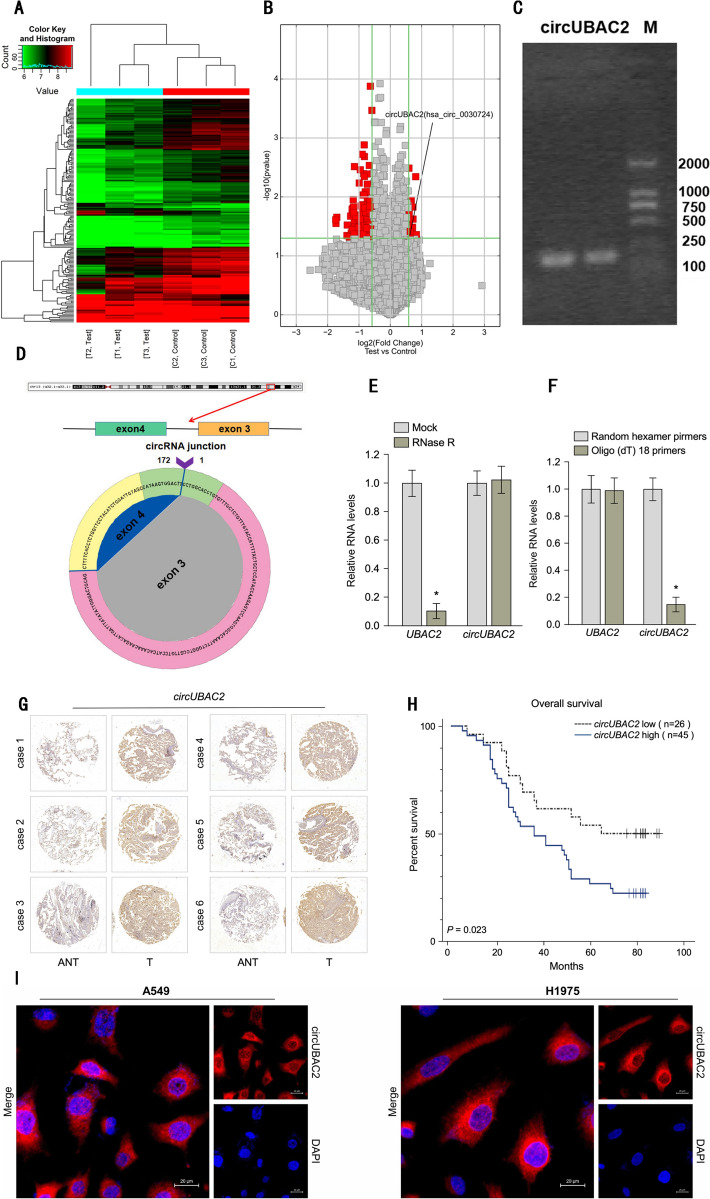
CircUBAC2 expression and identification. **A** Heat maps showing differentially expressed circRNAs in three lung adenocarcinoma samples (T, tumor) and adjacent normal tissues (C, control). **B** Volcano plots showing differences in circRNA expression levels between cancerous and paracancerous tissues. **C** Agarose gel electrophoresis detection of circUBAC2 in lung adenocarcinoma tissues. **D** Schematic diagram of circUBAC2 and its Sanger sequencing. **E** qRT-PCR detection of relative RNA levels following treatment with RNase R or mock among A549 cell-derived total RNAs. **F** The results of reverse transcription assays using random hexamer or oligo(dT)18 primers. qRT-PCR was employed to examine the relative RNA levels, followed by normalization to the levels generated using random hexamer primers. **G** RNA-ISH detection of circUBAC2 expression in lung adenocarcinoma tissues and adjacent tissues. **H** Overall survival of patients with lung adenocarcinoma with high or low circUBAC2 expression, as assessed using Kaplan–Meier analysis.** I** The subcellular localization of circUBAC2 in lung adenocarcinoma cells assessed using FISH. Data are presented as the means ± SDs; *P < 0.05

Microarray analysis of circRNAs in three lung adenocarcinoma tissue specimens and their adjacent noncancerous tissue was carried out. The differences in the expression levels of circRNAs between noncancerous and cancerous tissues were illustrated using volcano plots (Fig. 1A, B). We detected 13,552 circRNAs, among which 22 showed upregulation and 150 showed downregulation. Compared with adjacent noncancer tissues, the expression of circUBAC2 (hsa_circ:0030724) was significantly upregulated in cancer tissues. Agarose gel electrophoresis indicated the molecular weight of circUBAC2 (Fig. 1C). Analysis showed that circUBAC2 is derived from splicing of exons 3 and 4 of the *UBAC2* gene (encoding UBA domain containing 2). Sanger sequencing using divergent primers covering the predicted circRNA confirmed the junction sequence (Fig. 1D). To determine the cyclic characteristics of circUBAC2, we extracted total RNA from A549 cells and added RNaseR. Compared with linear *UBAC2* mRNA, circUBAC2 was more stable in the presence of RNaseR (Fig. 1E). Next, random hexamers and oligo(dT)18 primers were designed and used for qRT-PCR. Lower expression of circUBAC2 was observed using oligo(dT)18 primers compared with using random hexamer primers, while the expression of linear *UBAC2* mRNA was similar using both sets of primers (Fig. 1F). Table S1A shows the clinicopathological data of the patients who provided the tissue used to construct the microarrays. According to the RNA-ISH results, circUBAC2 expression was higher in cancer tissues from patients with lung adenocarcinoma compared with that in their adjacent noncancerous tissues (Fig. 1G). Among the patients, 63.3% showed high circUBAC2 expression in their tumors (Table S1B). There was no statistical correlation between circUBAC2 expression and the patients' lymph node metastasis status, distant metastasis status, and age; however, American Joint Committee on Cancer (AJCC) clinical stage and patient survival correlated significantly with circUBAC2 expression (Table 1). Subsequent Cox multivariate analysis confirmed the significant correlation between circUBAC2 expression and AJCC clinical stage. Thus, circUBAC2 expression could be utilized as an independent factor to assess patient prognosis (Table 2). Patients with high circUBAC2 expression had significantly shorter survival compared with those with low circUBAC2 expression (Fig. 1H). A FISH assay indicated the cytoplasmic location of circUBAC2 (Fig. 1I).

**Table 1:** Correlation between circUBAC2 expression and clinicopathological characteristics of lung cancer

Characteristics		circUBAC2		Chi-squared test *P*-value	Fisher’s exact test *P*-value
		Low no. cases	High no. cases		
Age (years)	> 55	16	30	0.663	0.797
	≤ 55	10	15		
AJCC clinical stage	I–II	15	40	0.002	0.006
	III–IV	11	5		
T classification	T1–T2	21	41	0.207	0.272
	T3–T4	5	4		
N classification	N0	13	30	0.166	0.210
	N1–N3	13	15		
M classification	No	26	45		
	Yes	0	0		
Gender	Male	13	27	0.413	0.463
	Female	13	18		
Survival or mortality	Survival	13	10	0.016	0.020
	Mortality	13	35

**Table 2:** Univariate and multivariate analyses of various prognostic parameters in patients with lung cancer by Cox regression analysis

	Univariate analysis			Multivariate analysis		
	No. patients	*P*-value	Relative risk	*P*-value	Relative risk	95% confidence interval
T stage						
T1 and T2	62	0.004	3.158	0.000	5.115	2.156–12.131
T3 and T4	9					
Expression of circUBAC2						
Low expression	26	0.028	2.051	0.004	2.759	1.398–5.588
High expression	45					

### The proliferation, invasion, and migration of lung adenocarcinoma cells was promoted by circUBAC2 in vitro

**Fig. 2:**
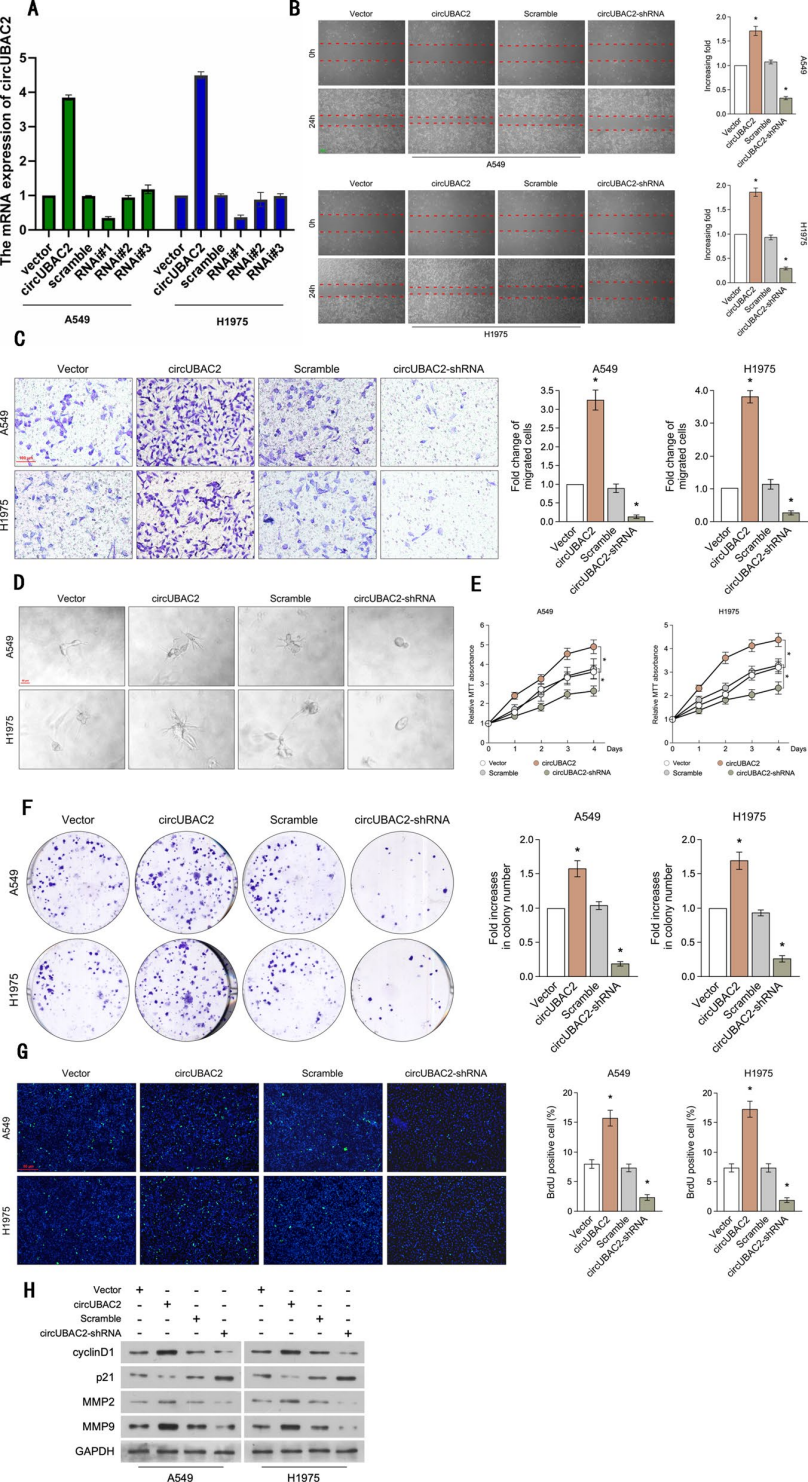
circUBAC2 promotes lung cancer cell proliferation, invasion, and migration in vitro. **A** qRT-PCR detection of circUBAC2 expression following cell transfection. **B** Wound healing assays of lung adenocarcinoma cell migration following cell transfection. **C** Transwell assays of lung adenocarcinoma cell invasion ability following transfection of the circUBAC2 overexpression or knockdown vectors. **D** Three-dimensional (3D) spheroid invasion assays of lung adenocarcinoma cell antennae growth in response to circUBAC2. **E** MTT assays detecting lung adenocarcinoma cell proliferation following transfection of circUBAC2 overexpression or knockdown vectors. **F** Colony formation assays of lung adenocarcinoma cell proliferation following transfection with circUBAC2 overexpression or knockdown vectors. **G** EdU immunofluorescence assays of lung adenocarcinoma cell proliferation. **H** Western blotting detection of the expression of cyclinD1, p21, MMP2, and MMP9 in lung adenocarcinoma cells transfected with circUBAC2 overexpression or knockdown vectors. Data are presented as the means ± SD; *P < 0.05

Four lentiviral vectors were transfected into adenocarcinoma cells (circUBAC2 overexpression vector [circUBAC2], circUBAC2 knockdown vector [circUBAC2-shRNA], empty vector [vector], and negative control vector [scramble]) to assess circUBAC2's effect on lung adenocarcinoma cells. Transfection efficiency into lung adenocarcinoma cells was assessed using qRT-PCR (Fig. 2A). Wound healing assays to detect the effect of circUBAC2 on the migration ability of lung adenocarcinoma cells showed that circUBAC2 overexpression increased lung adenocarcinoma cell migration. By contrast, the migration ability of lung adenocarcinoma cells was weakened after circUBAC2 knockdown (Fig. 2B). Transwell assays of lung adenocarcinoma cell invasion indicated that circUBAC2 overexpression enhanced cell invasion, while circUBAC2 knockdown had the opposite effect (Fig. 2C). Furthermore, three-dimensional (3D) spheroid invasion assays also suggested that circUBAC2 promotes lung adenocarcinoma cell invasion (Fig. 2D). Next, MTT assays, colony formation assays, and EdU immunofluorescence assays were used to assess cell proliferation. The MTT assays showed a positive correlation between circUBAC2 expression and lung adenocarcinoma cell proliferation (Fig. 2E). In the clone formation assays, circUBAC2 was observed to increase the number of spheroids, thus promoting the proliferation of lung adenocarcinoma cells (Fig. 2F). The EdU immunofluorescence assays demonstrated the same trend (Fig. 2G). In addition, western blotting showed that circUBAC2 overexpression increased the levels of cyclinD1, matrix metalloproteinase (MMP)2, and MMP9, but decreased the levels of p21. The opposite trend was observed after knocking down circUBAC2 (Fig. 2H). Altogether, these findings indicated that circUBAC2 enhanced lung adenocarcinoma cell proliferation, invasion, and migration.

### circUBAC2 promoted the progression of lung adenocarcinoma in vivo

**Fig. 3:**
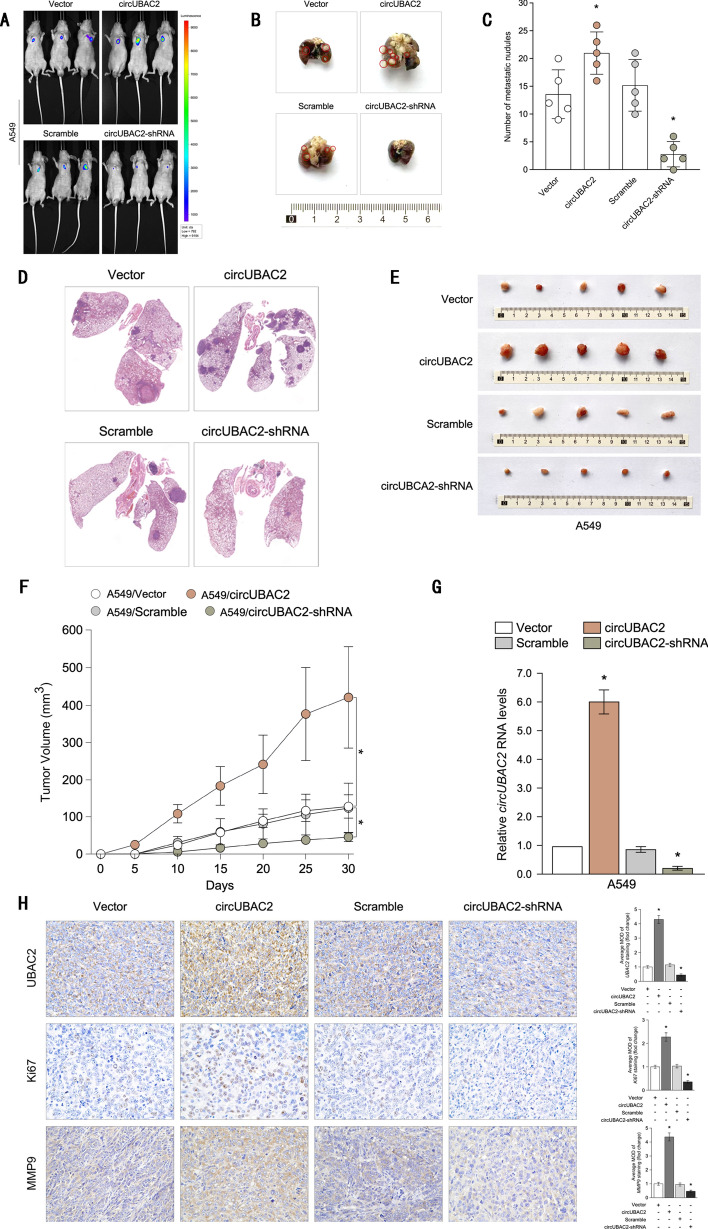
circUBAC2 promotes lung adenocarcinoma cell metastasis and proliferation in vivo. **A** The luminescence intensity in the mouse tail vein tumor metastasis model. **B** Images of lung tissue in the mouse tail vein tumor metastasis model. **C** Statistical analysis of metastatic lung nodules. **D** HE staining of lung tissues. **E** Image of xenograft subcutaneous tumors. **F** Tumor volume graph for the xenograft subcutaneous tumor model. **G** qRT-PCR detection of circUBAC2 expression in mouse tumor tissues. **H** Immunohistochemistry analysis of UBAC2, Ki67, and MMP9 expression in mouse tumor tissues. Data are presented as the means ± SD; *P < 0.05

We evaluated the role of circUBAC2 in nude mice. Four lentiviral vectors (circUBAC2 overexpression vector, circUBAC2 knockdown vector, empty vector, and negative control vector) were transfected into A549 cells. To observe lung metastasis, we injected stably transfected A549 cells into mouse tail veins. First, we carried out intravital fluorescence imaging, followed by sacrifice of the mice and examination of metastatic tumor nodule formation in the lungs. The results showed that the mice receiving cells overexpressing circUBAC2 had more metastatic nodules compared with the mice receiving cells with circUBAC2 knockdown (Fig. 3A–D). Tumor growth was assessed by subcutaneously injecting BALB/c nude mice with the above cells. The mice receiving cells overexpressing circUBAC2 showed a rapid increase in tumor volume and weight, while the tumor volume and weight of the mice receiving cells with circUBAC2 knockdown were relatively smaller (Fig. 3E, F). qRT-PCR was used to validate circUBAC2 expression in mouse tumor tissue (Fig. 3G). According to immunohistochemistry, circUBAC2 expression correlated positively with Ki67 (a marker of proliferation) and MMP9 expression in mouse tumors (Fig. 3H).

### circUBAC2 inactivated the Hippo signaling pathway

**Fig. 4:**
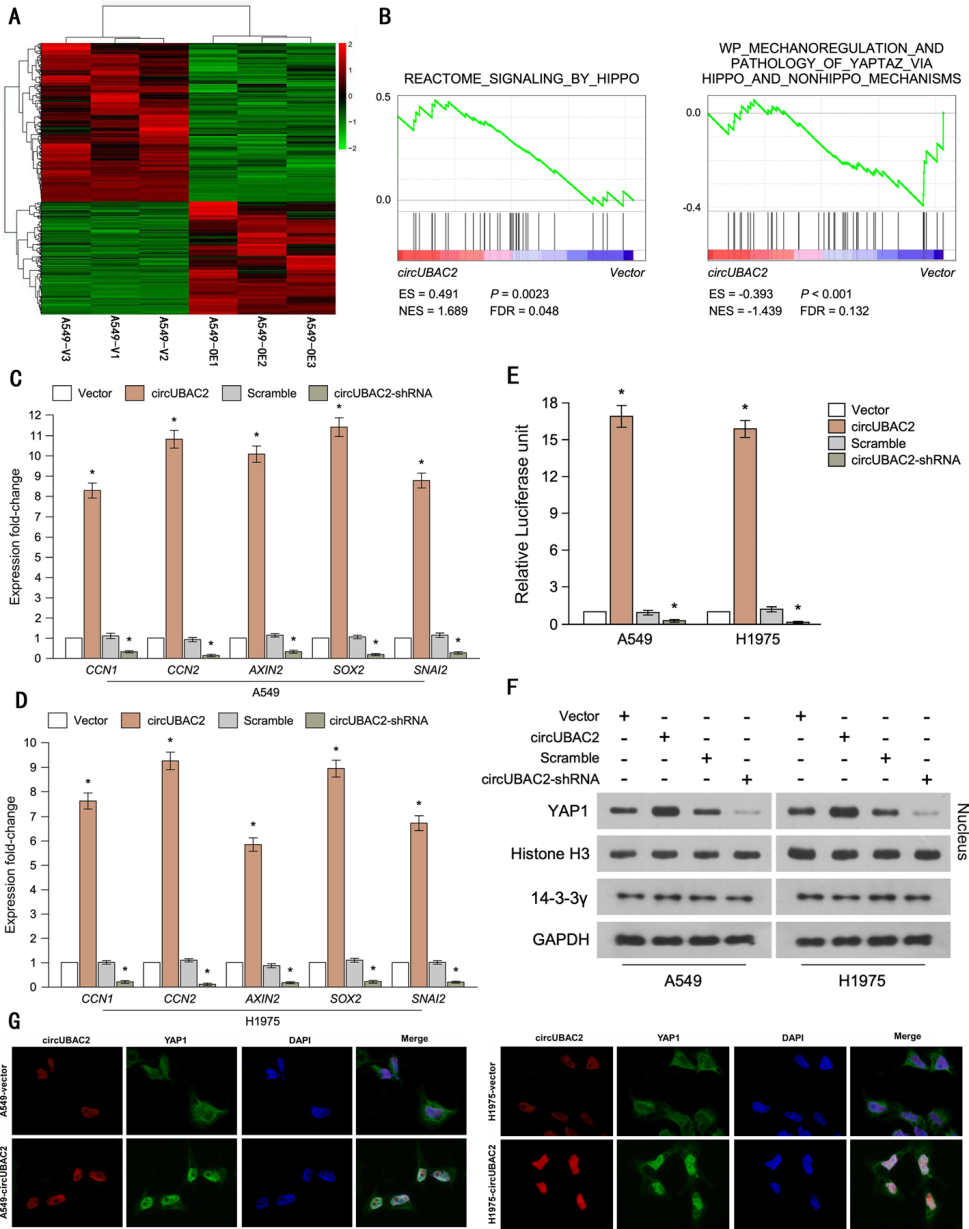
circUBAC2 inactivates the Hippo signaling pathway. **A** Transcriptome sequencing results revealing differences in gene expression. **B** GSEA results showing a correlation between circUBAC2 and the Hippo signaling pathway. **C**, **D** qRT-PCR detection of CCN1, CCN2, AXIN2, SOX2, and SNAI2 expression. **E** Luciferase activity of TEAD following transfection of the circUBAC2 knockdown or overexpression vector. **F** Western blotting detection of 14-3-3-γ and YAP1 expression in lung adenocarcinoma cells after transfection of the circUBAC2 knockdown and overexpression vectors. **G** Immunofluorescence assays detecting the localization of circUBAC2 and YAP in cells after transfection of the control vector or the circUBAC2 overexpression vector. Data are presented as the means ± SD; *P < 0.05

To search for the downstream targets of circUBAC2, we performed mRNA sequencing on A549 cells transfected with the circUBAC2 overexpression vector. Figure 4A shows the differential gene expression heat map generated from the transcriptome data (Fig. 4A). Gene Set Enrichment Analysis (GSEA) indicated a significant correlation between the Hippo signaling pathway and circUBAC2 expression (Fig. 4B). Uniquely, inactivation of the Hippo signaling pathway can lead to tumorigenesis. Cancer cell malignancy, invasion, and migration are promoted by mutations or altered expression levels of the core pathway components (macrophage stimulating [MST]1/2, large tumor suppressor kinase [LATS]1/2, YAP, and WW domain containing transcription regulator 1 [TAZ]) [18]. The expression levels of cellular communication network factor 1 (CCN1), CCN2, axin 2 (AXIN2), SRY-box transcription factor 2 (SOX2), and snail family transcriptional repressor 2 (SNAI2) are directly regulated by the Hippo signaling pathway [19, 20]. The expression of these genes is regulated by TEA domain transcription factor (TEAD) [21, 22]. YAP is an effector of the Hippo signaling pathway. When the Hippo signaling pathway was inactivated, YAP entered the nucleus to activate TEAD transcription [23]. To explore the impact of circUBAC2 on the Hippo signaling pathway, we examined the expression levels of CCN1, CCN2, AXIN2, SOX2, and SNAI. The results showed that circUBAC2 expression correlated positively with the expression of CCN1, CCN2, AXIN2, SOX2, and SNAI (Fig. 4C, D). The activation of TEAD after overexpression or knockdown of circUBAC2 was detected using luciferase activity assays, which showed significantly enhanced luciferase activity in the circUBAC2 overexpression group relative to the control group. We observed that the luciferase activity of the circUBAC2-knockdown group was significantly reduced (Fig. 4E). The 14-3-3 family of proteins (tyrosine 3-monooxygenase/tryptophan 5 monooxygenase activation protein [14-3-3-ε, 14-3-3-γ, and 14-3-3-η]) can bind to YAP to prevent its entry into the nucleus where it exerts its effects [24]. Western blotting revealed that the expression of circUBAC2 in lung adenocarcinoma cells promotes YAP nuclear localization and reduces the expression of YAP in the cytoplasm (Fig. 4F; Supplementary Fig. 4A). However, the expression of 14-3-3-γ did not appear to be regulated by circUBAC2 (Fig. 4F). Immunofluorescence analysis showed that circUBAC2 promoted, whereas circUBAC2 deficiency inhibited, YAP entry into the nucleus (Fig. 4G). These results confirmed that circUBAC2 inactivates the Hippo signaling pathway.

### circUBAC2 inhibited YAP–14-3-3-γ binding to trigger YAP nuclear localization

**Fig. 5:**
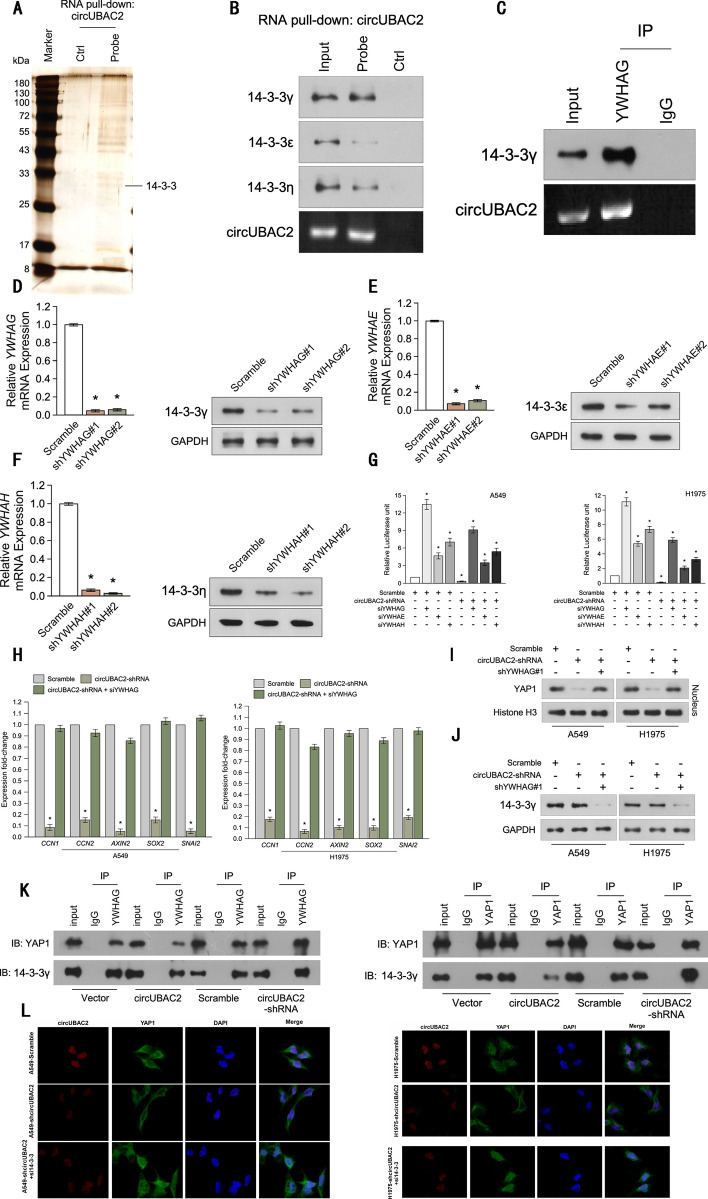
circUBAC2 inhibited YAP–14-3-3-γ binding to trigger YAP nuclear localization. **A** Analysis of proteins derived from RNA pull-down using the circUBAC2 probe and negative control assessed by silver-stained SDS–PAGE. The identified protein was subjected to mass spectrometry analysis. **B** RNA pull-down assays detecting the binding of circUBAC2 with 14-3-3-ε, 14-3-3-η, and 14-3-3-γ. **C** RIP detecting the binding of circUBAC2 with 14-3-3-γ. **D** qRT-PCR and western blotting detection of 14-3-3-γ expression following transfection of the YWHAG knockdown vector. **E** qRT-PCR and western blotting detection of YWHAE /14-3-3-ε expression following transfection of the YWHAE knockdown vector. **F** qRT-PCR and western blotting detection of YWHAH/14-3-3-η expression following transfection of the YWHAH knockdown vector. **G** The luciferase activity of TEAD following co-transfection of the circUBAC2 knockdown vector and the YWHAE/YWHAG/YWHAH knockdown vector. **H** qRT-PCR detection of CCN1, CCN2, AXIN2, SOX2, and SNAI2 expression in lung adenocarcinoma cells after co-transfection of the circUBAC2 knockdown vector and the YWHAE/YWHAG/YWHAH knockdown vector. **I** Western blot detection of 14-3-3-γ expression in lung adenocarcinoma cells after co-transfection of the circUBAC2 knockdown vector and the YWHAG knockdown vector. **J** Western blot detection of YAP1 expression in lung adenocarcinoma cells after co-transfection of the circUBAC2 knockdown vector and the YWHAG knockdown vector. **K** Immunoprecipitation assays detecting the effect of circUBAC2 on the 14-3-3-γ/YAP complex. **L** Immunofluorescence assays detecting the localization of circUBAC2 and YAP in cells after co-transfection of the circUBAC2 knockdown vector and the YWHAE/YWHAG/YWHAH knockdown vector. Data are presented as the means ± SD; *P < 0.05

Recent studies reported that circular RNAs could function by interacting with proteins; however, the specific mechanisms are still unclear (reviewed in ref. [25]). To identify the downstream target proteins of circUBAC2, we constructed circUBAC2 with an F2 tag. Then, we transfected the vector into lung adenocarcinoma cells, precipitated the target protein using RNA pull-down assays, and carried out protein mass spectrometry analysis. The results indicated that circUBAC2 might combine with 14-3-3-ε, 14-3-3-γ, and 14-3-3-η (Fig. 5A). Further western blotting and RNA pull-down assays confirmed this result (Fig. 5B). RNA pull-down assays showed that UBAC2 could not bind to 14-3-3-ε, 14-3-3-γ, and 14-3-3-η (Supplementary Fig. 5).To explore the regulatory mechanism of circUBAC2 on 14-3-3-ε, 14-3-3-γ, and 14-3-3-η, YWHAG/YWHAE/YWHAH knockdown vectors (YWHAG/shRNA, YWHAE/shRNA, YWHAH/shRNA) and circUBAC2 knockdown vector were cotransfected into lung adenocarcinoma cells. Western blotting and qRT-PCR confirmed the transfection efficiency (Fig. 5D–F). Luciferase assays detected the combined effects of circUBAC2 and 14-3-3-ε/ 14-3-3-γ/14-3-3-η on TEAD transcription activity. Knockdown of circUBAC2 inhibited the luciferase activity, while YWHAE/YWHAG/YWHAH knockdown activated luciferase activity. When the cells were co-transfected with both circUBAC2 and YWHAE/YWHAG/YWHAH knockdown vectors, YWHAE/YWHAG/YWHAH deficiency neutralized the effects caused by circUBAC2 deficiency (Fig. 5G). The results of RIP analysis showed that circUBAC2 could bind 14-3-3-γ (Fig. 5C). qRT-PCR was then used to detect the expression of CCN1, CCN2, AXIN2, SOX2, and SNAI2. The results indicated that circUBAC2 deficiency inhibited the expression of CCN1, CCN2, AXIN2, SOX2, and SNAI2 at the RNA level, while YWHAG deficiency reversed this trend (Fig. 5H). Western blotting showed that circUBAC2 deficiency decreased YAP1 protein levels in the nucleus, while YWHAG knockdown reversed this trend (Fig. 5I). circUBAC2 deficiency enhanced YAP1 protein levels in the cytoplasm, while YWHAG knockdown reversed this trend (Supplementary Fig. 4B). However, the expression of 14-3-3-γ was not regulated by circUBAC2 (Fig. 5J). Therefore, we believed that circUBAC2 could affect the localization of YAP1 without affecting the expression of 14-3-3-γ. To interrogate the hypothesized competitive binding between circUBAC2 and YAP for 14-3-3-γ, co-immunoprecipitation assays were performed using anti-14-3-3-γ antibodies under endogenous expression conditions. After pulling down YAP using the anti-14-3-3-γ antibody, western blotting showed that circUBAC2 hindered the binding of 14-3-3-γ to YAP, while circUBAC2 deficiency promoted the binding of 14-3-3-γ to YAP. Using the anti-YAP antibody to pull down 14-3-3-γ obtained the same results (Fig. 5K). The results of immunofluorescence experiments showed that circUBAC2 deficiency inhibited the nuclear localization of YAP, while YWHAG deficiency reversed this trend (Fig. 5L).

### circUBAC2 promoted lung adenocarcinoma cells progression by regulating the 14-3-3-γ/YAP axis

**Fig. 6:**
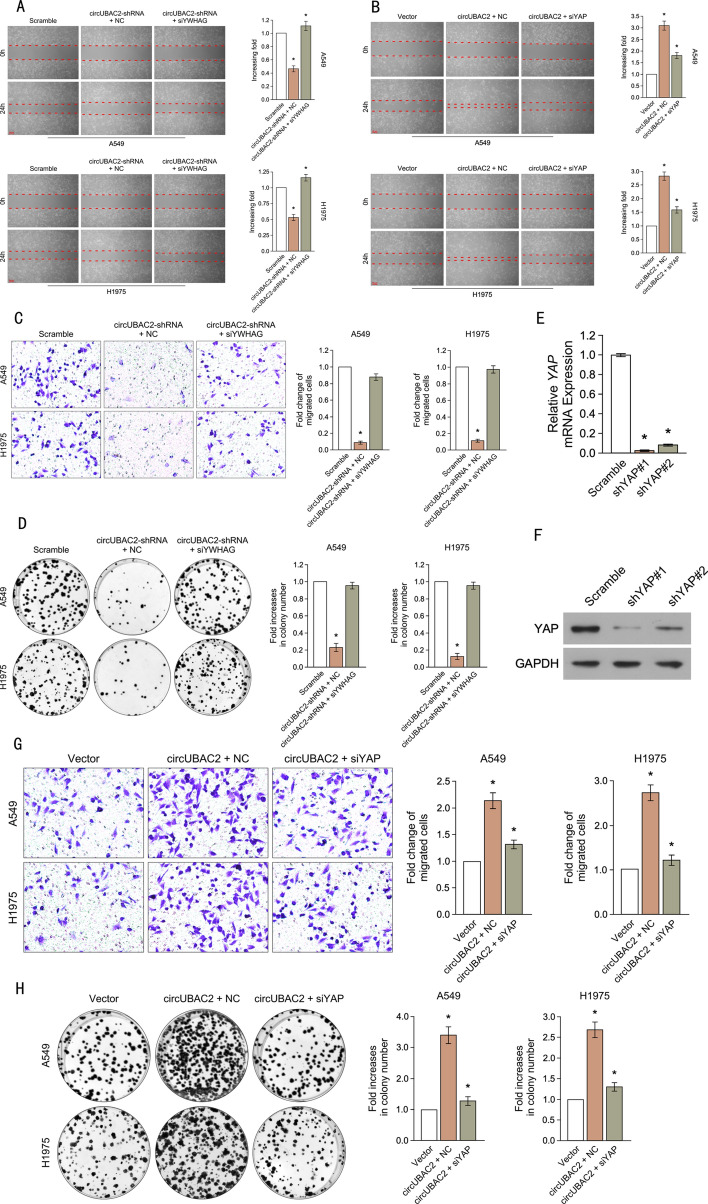
circUBAC2 promotes lung adenocarcinoma progression by regulating the 14-3-3-γ/YAP axis. **A** Wound healing assays examining lung adenocarcinoma cell migration after co-transfection of the circUBAC2 knockdown vector and YWHAG knockdown vector. **B** Wound healing assays examining lung adenocarcinoma cell migration following co-transfection of the circUBAC2 overexpression vector and the YAP knockdown vector. **C** Transwell assays examining lung adenocarcinoma cell invasion after co-transfection of the circUBAC2 knockdown vector and the YWHAG knockdown vector. **D** Colony formation assays examining lung adenocarcinoma cell proliferation following co-transfection of the circUBAC2 knockdown vector and the YWHAG knockdown vector. **E**, **F** qRT-PCR and western blot detection of the expression of YAP following transfection of the YAP knockdown vector. **G** Transwell assays examining lung adenocarcinoma cell invasion after co-transfection of the circUBAC2 overexpression vector and the YAP knockdown vector. **H** Colony formation assays examining the proliferative capacity of lung adenocarcinoma cell proliferation following co-transfection of the circUBAC2 overexpression vector and the YAP knockdown vector. Data are presented as the means ± SD; *P < 0.05

To explore circUBAC2's effects on Hippo signaling, YWHAG and YAP knockdown vectors were constructed. Upon co-transfection of the YWHAG knockdown vectors and circUBAC2 knockdown vectors into lung adenocarcinoma cells, wound healing assays suggested that circUBAC2 deficiency inhibited the migration of lung adenocarcinoma cells, while YWHAG deficiency reversed this trend (Fig. 6A). These results were also validated using Transwell assays (Fig. 6C). circUBAC2 deficiency suppressed the proliferation of lung adenocarcinoma cells, while YWHAG deficiency weakened this trend (Fig. 6D). The knockdown efficiency of YAP was verified through qRT-PCR and western blotting analysis (Fig. 6E, F). The YAP knockdown vector and circUBAC2 overexpression vector were co-transfected into lung adenocarcinoma cells. circUBAC2 could facilitate the migration and invasion of lung adenocarcinoma cells; however, in the co-transfected lung adenocarcinoma cells, this trend was reversed (Fig. 6B, G). The results of the colony formation assays showed that circUBAC2 promoted the proliferation of lung adenocarcinoma cells; however, this effect was attenuated after simultaneously transfecting the YAP knockdown vector and the circUBAC2 overexpression vector (Fig. 6H). Therefore, our results suggested that circUBAC2 promoted the progression of lung adenocarcinoma cells by regulating the 14-3-3-γ/YAP axis.

### circUBAC2 facilitated OTUB1–YAP interaction to trigger YAP deubiquitination and enhance its stability

**Fig. 7:**
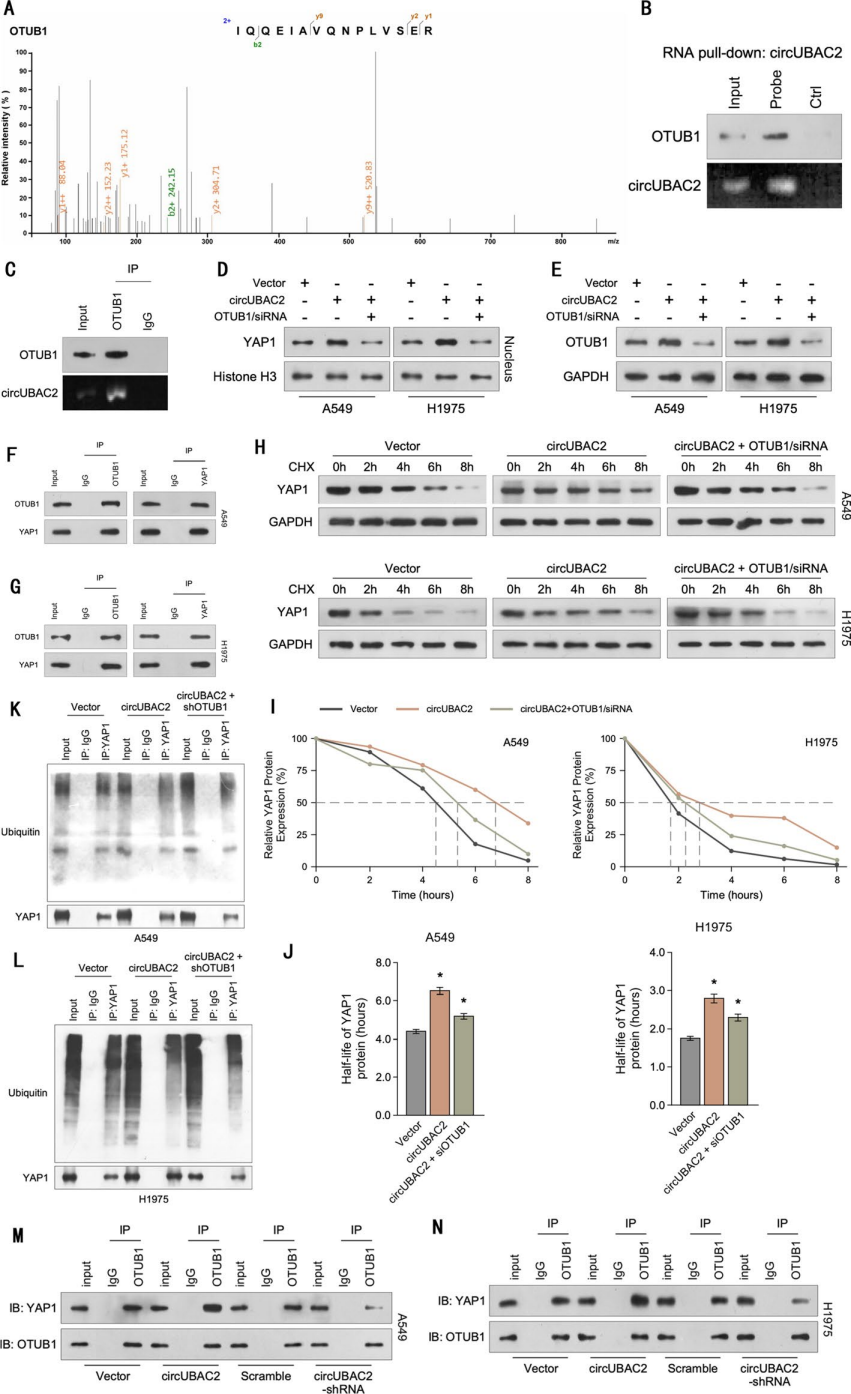
circUBAC2 facilitated OTUB1–YAP interactions to trigger YAP deubiquitination and enhance its stability. **A** Mass spectrometry analysis of circUBAC2-bound proteins isolated by RNA pull-down revealed the presence of the OTUB1 peptide peak. **B** RNA pull-down assays were performed to detect the binding of circUBAC2 to OTUB1. **C** The binding of circUBAC2 to OTUB1 was detected by RIP assays. **D**, **E** Western blotting was performed to detect the expression of OTUB1 and nuclear YAP in lung adenocarcinoma cells following co-transfection with the circUBAC2 overexpression vector and OTUB1 knockdown vector. **F**, **G** Co-immunoprecipitation was performed to detect the interaction between OTUB1 and YAP. **H**–**J** YAP protein half-life was measured by cycloheximide chase assay in cells co-transfected with circUBAC2 overexpression plasmid and OTUB1 siRNA. **K**, **L** The effect of circUBAC2 on YAP ubiquitination was assessed by ubiquitination assay following co-transfection with the circUBAC2 overexpression vector and the OTUB1 knockdown vector. **M**, **N** Immunoprecipitation assays were performed to assess the effect of circUBAC2 on the OTUB1/YAP complex. Data are presented as the means ± SD; *P < 0.05

After conducting RNA pull-down and mass spectrometry screening on circUBAC2-related proteins in the previous section, we further discovered that OTUB1 may interact with circUBAC2 (Fig. 7A). Subsequent RNA pull-down and RIP assays confirmed its direct binding to circUBAC2 (Fig. 7B, C). OTUB1, a member of the OTU deubiquitinase family, removes ubiquitin chains from substrate proteins to stabilize them and modulate their activity in cellular signaling pathways [26]. In gastric cancer, OTUB1 is upregulated and drives tumor proliferation by suppressing Hippo signaling to activate oncogenic YAP/TAZ. OTUB1 mediates YAP deubiquitination, thereby stabilizing YAP [27]. To investigate the role of OTUB1 in lung cancer cells, OTUB1 expression was knocked down using small interfering RNA (siRNA). Western blot analysis demonstrated that circUBAC2 enhanced nuclear YAP expression, whereas OTUB1 deficiency reduced YAP levels in the nucleus (Fig. 7D). circUBAC2 attenuated the expression of YAP in the cytoplasm, while the absence of OTUB1 reversed this trend (Supplementary Fig. 4C). Western blot analysis was conducted to assess the efficiency of OTUB1 deficiency (Fig. 7E). Endogenous co-immunoprecipitation assays demonstrated that OTUB1 interacts with YAP in lung cancer cells (Fig. 7F, G). The protein half-life assay revealed that circUBAC2 enhanced YAP protein stability in lung cancer cells, an effect that was reversed by OTUB1 depletion (Fig. 7H–J). circUBAC2-shRNA promoted the degradation of YAP protein (Supplementary Fig. 3A–C). To verify whether circUBAC2 enhances the interaction between OYUB1 and YAP, we performed co-immunoprecipitation (Co-IP) assays. Immunoprecipitation of YAP using an anti-OTUB1 antibody, followed by western blotting, revealed that circUBAC2 overexpression strengthened the OTUB1–YAP binding, whereas circUBAC2 knockdown attenuated this interaction (Fig. 7M, N). Ubiquitination assays revealed that circUBAC2 overexpression reduced YAP ubiquitination, whereas OTUB1 knockdown reversed this effect (Fig. 7K, L).

### circUBAC2 facilitated lung adenocarcinoma progression by modulating the OTUB1–YAP axis

**Fig. 8:**
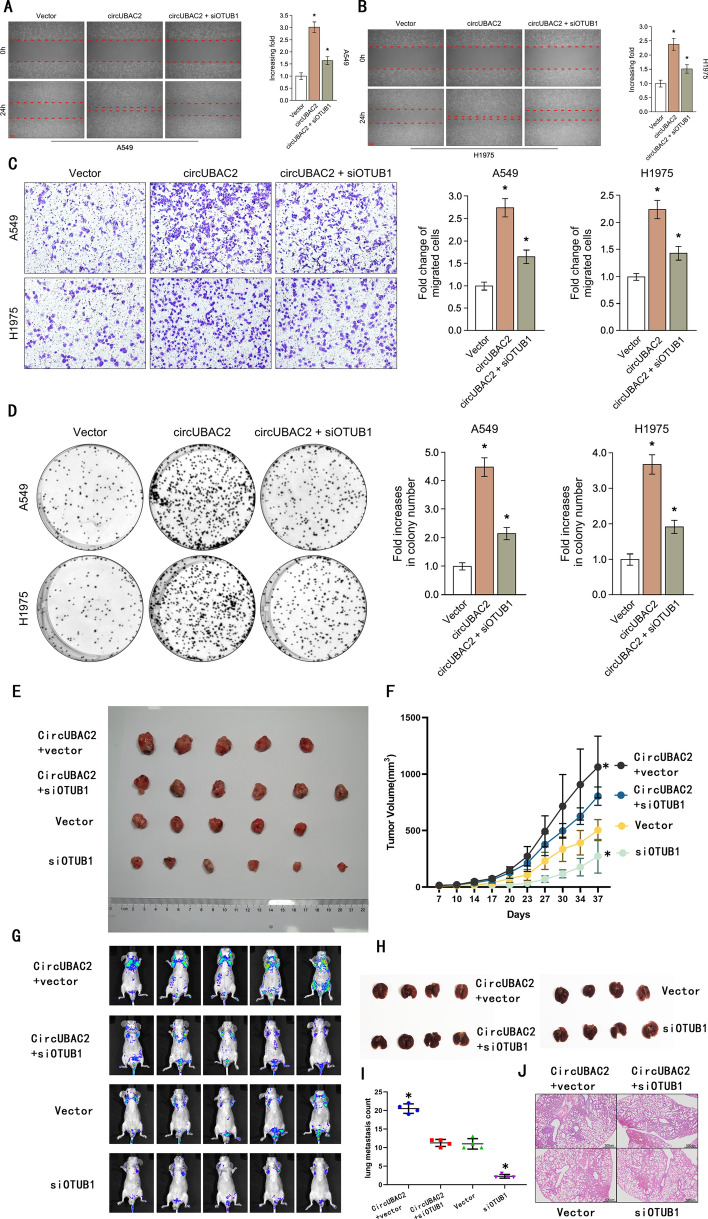
circUBAC2 facilitated lung adenocarcinoma progression by modulating the OTUB1–YAP axis. **A**, **B** Wound healing assays were performed to assess lung adenocarcinoma cell migration following co-transfection with the circUBAC2 overexpression vector and OTUB1 knockdown vector. **C** Transwell assays were conducted to evaluate lung adenocarcinoma cell invasion following co-transfection with the circUBAC2 overexpression vector and OTUB1 knockdown vector. **D** Colony formation assays were performed to evaluate lung adenocarcinoma cell proliferation following co-transfection with the circUBAC2 overexpression vector and OTUB1 knockdown vector. **E** Subcutaneous xenograft models were established using lung adenocarcinoma cells co-transfected with the circUBAC2 overexpression vector and OTUB1 knockdown vector to assess tumor growth in vivo. **F** Quantification of tumor growth kinetics in subcutaneous xenograft models. **G** Quantitative bioluminescence imaging was performed to assess metastatic burden in tail-vein-injected tumor models following co-transfection of circUBAC2 overexpression and OTUB1 knockdown vectors. **H**, **I** Quantitative assessment of metastatic lung nodule burden. **J** Histopathological evaluation of lung tissue sections was conducted using hematoxylin and eosin (HE) staining to assess metastatic colonization. Data are presented as the means ± SD; *P < 0.05

To examine the biological role of OTUB1 in lung cancer cells, the circUBAC2 overexpression vector and the OTUB1 knockdown vector were transfected into the cells. Wound healing assays demonstrated that circUBAC2 promoted lung cancer cell migration, whereas OTUB1 knockdown attenuated this pro-migratory effect (Fig. 8A, B). Transwell assays revealed that circUBAC2 promoted cell invasion, an effect that was diminished by OTUB1 knockdown (Fig. 8C). The colony formation results indicated that circUBAC2-mediated proliferative advantage was counteracted by OTUB1 depletion (Fig. 8D). To assess the biological function of OTUB1 in vivo, we employed two complementary mouse models: (1) a subcutaneous xenograft tumor model in nude mice to evaluate tumor growth, and (2) a tail vein injection metastasis model to examine lung colonization capacity. In the nude mouse xenograft model, OTUB1 knockdown significantly inhibited tumor growth compared with the control group. Consistent with its oncogenic role, circUBAC2 overexpression promoted tumor progression, yielding larger tumors than controls. Notably, co-knockdown of OTUB1 in circUBAC2-overexpressing cells attenuated this growth-promoting effect (Fig. 8E, F). To evaluate lung metastasis, we intravenously injected stably transfected A549 cells into nude mice via the tail vein. Metastatic progression was monitored using in vivo fluorescence imaging, followed by necropsy and quantification of pulmonary tumor nodules. Quantitative analysis demonstrated that OTUB1 knockdown significantly reduced the number of metastatic lung nodules compared with control mice. Importantly, in circUBAC2-overexpressing cells, OTUB1 depletion further attenuated metastatic potential, resulting in significantly fewer pulmonary nodules relative to circUBAC2 overexpression alone (Fig. 8G–J).

### EIF4A3-induced circUBAC2 promoted lung adenocarcinoma progression

**Fig. 9:**
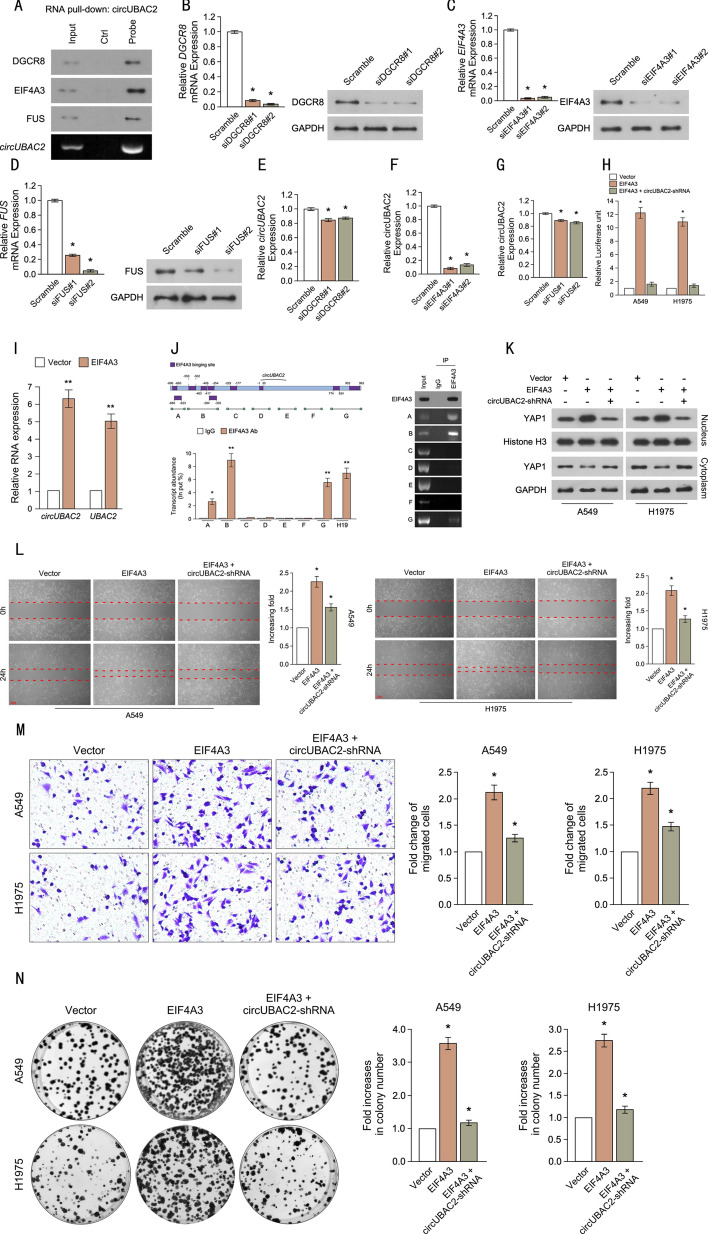
EIF4A3-induced circUBAC2 promoted lung adenocarcinoma progression. **A** RNA pull-down assays to detect the binding of circUBAC2 with DGCR8, EIF4A3, and FUS. **B** qRT-PCR and western blotting detection of DGCR8 expression following transfection of the DGCR8 knockdown vector. **C** qRT-PCR and western blotting detection of EIF4A3 expression following transfection of the EIF4A3 knockdown vector. **D** qRT-PCR and western blotting detection of FUS expression following transfection of the FUS knockdown vector. **E** qRT-PCR detection of circUBAC2 expression following transfection of the DGCR8 knockdown vector. **F** qRT-PCR detection of circUBAC2 expression following transfection of the EIF4A3 knockdown vector. **G** qRT-PCR detection of circUBAC2 expression following transfection of the FUS knockdown vector. **H** Detection the luciferase activity of TEAD following co-transfection of the circUBAC2 knockdown vector and EIF4A3 overexpression vector. **I** qRT-PCR detection of circUBAC2 and *UBAC2* mRNA expression following transfection of the EIF4A3 overexpression vector. **J** Chromosome co-precipitation to determine the binding site between the *UBAC2* mRNA precursor and EIF4A3. **K** Western blotting detection of the levels of YAP1 (nucleus) and YAP1 (cytoplasm) after co-transfection of the circUBAC2 knockdown vector and EIF4A3 overexpression vector. **L** Wound healing assays examining lung adenocarcinoma cell migration after co-transfection of the circUBAC2 knockdown vector and EIF4A3 overexpression vector. **M** Transwell assays examining lung adenocarcinoma cell invasion after co-transfection of the circUBAC2 knockdown vector and EIF4A3 overexpression vector. **N** Colony formation assays of lung adenocarcinoma cell proliferation after co-transfection of the circUBAC2 knockdown vector and EIF4A3 overexpression vector. Data are presented as the means ± SD; *P < 0.05

RNA binding proteins (RBPs) regulate circRNA biogenesis from exons [28]. To investigate the mechanism by which RBPs regulate circUBAC2 expression, the CircInteractome database was searched, which identified potential RBP binding sites in circUBAC2. EIF4A3, DiGeorge syndrome critical region gene 8 (DGCR8), and fused in sarcoma (FUS) were found to be the most likely RBPs to bind to circUBAC2. EIF4A3, DGCR8, and FUS are modulators of circRNA back splicing [29, 30]. RNA pull-down assays found that EIF4A3 was the most significantly enriched by circUBAC2 (Fig. 9A). We constructed DGCR8, EIF4A3, and FUS knockdown plasmid vectors (DGCR8/siRNA, EIF4A3/siRNA, FUS/siRNA). After transfecting these plasmid vectors into A549 cells, we tested the efficiency of transfection (Fig. 9B–D). qRT-PCR detection of circUBAC2 expression showed a positive correlation between the expression of circUBAC2 and the expression of *EIF4A3*, *FUS*, and *DGCR8*. Among them, the EIF4A3 knockdown resulted in the most significant difference in circUBAC2 expression (Fig. 9E–G). Following transfection of the *EIF4A3* overexpression vector, the luciferase activity of TEAD in lung adenocarcinoma cells was enhanced. However, simultaneous knockdown of circUBAC2 reversed these results (Fig. 9H). Further qRT-PCR analysis demonstrated that EIF4A3 upregulated both circUBAC2 and *UBAC2* mRNA expression (Fig. 9I). Subsequently, ChIP assays ware used to detect the specific binding sites of EIF4A3. Using the anti-EIF4A3 antibody, EIF4A3 was demonstrated to bind to the *UBAC2* pre-mRNA via the putative binding sites, but not when the negative control antibody IgG was used (Fig. 9J). All the results suggested that EIF4A3 could bind to the 3′ start flanking intron region of pre-UBAC2 to promote the back splicing of circUBAC2. Western blotting analysis showed that EIF4A3 promoted the transfer of YAP from the cytoplasm to the nucleus. However, circUBAC2 knockdown reversed the above results (Fig. 9K). The EIF4A3 overexpression construct and the circUBAC2 knockdown construct were cotransfected into lung adenocarcinoma cells, and the proliferation, invasion, and migration of the cells were determined. Compared with the control group, EIF4A3 promoted the migration of lung adenocarcinoma cells. However, knockdown of circUBAC2 reversed this trend when the EIF4A3 overexpression vector and the circUBAC2 knockdown vector were cotransfected (Fig. 9L). Cell invasion assays confirmed this trend (Fig. 9M). Colony formation assays indicated that EIF4A3 enhanced the proliferation of lung adenocarcinoma cells, whereas knockdown of circUBAC2 neutralized this ability (Fig. 9N). Therefore, these results confirmed that EIF4A3 can exert its biological effects by regulating the expression of circUBAC2.

## Discussion

Lung cancer occurrence and development correlate highly with its pathological type [5], among which lung adenocarcinoma is a major pathological type. Traditional chemotherapy can no longer meet the needs of patients with lung cancer. However, the emergence of targeted therapy and immunotherapy has brought new hope to these patients [31]. Unfortunately, such treatments cannot benefit all patients with lung cancer. The emergence of side effects and drug resistance has also cast a shadow over lung cancer treatment [32]. Consequently, further research into the mechanisms of lung cancer occurrence and development is required.

With the emergence of new molecular biology methods, circRNAs have been found to be widely involved in the progression of various human diseases. Increasing evidence suggests the important roles played by circRNAs in cancer progression [33]. Initially, circRNAs were discovered to serve as molecular sponges for miRNAs, exerting their biological effects by regulating miRNA function [34]. Previously, we confirmed that hsa_circ_0021727 promotes the progression of esophageal squamous cell carcinoma by targeting miR-23b-5p. There are similar reports in lung cancer. For example, circHERC1 sequesters forkhead box O1 (FOXO1) in the cytoplasm by regulating the miR-142-3p–HMGB1 axis, thereby promoting the progression of non-small cell lung cancer [35]. circRNAs were once considered to be noncoding RNA. However, recently, certain circRNAs were confirmed to encode peptides with biological functions. circEMA4B can inhibit the progression of breast cancer by encoding peptide SEMA4B-11aa [36]. circSHPRH encodes SHPRH-146aa, which acts as a negative regulatory factor in glioblastoma [37]. circRNAs can also participate in protein regulation. circRNA-CREIT functions as a scaffold that aids the interaction between the E3 ligase HECT domain and ankyrin repeat containing E3 ubiquitin protein ligase 1 (HACE1) and protein kinase R (PKR) to enhance PKR proteasomal degradation via K48-linked polyubiquitylation [38]. circMYBL2 increased the binding of *FLT3* mRNA (encoding Fms related receptor tyrosine kinase 3) to polypyrimidine tract-binding protein 1 (PTBP1) to enhance FLT3’s translational efficiency [39]. In this study, we identified that a new circRNA, circUBAC2, was highly expressed in lung cancer tissue and correlated negatively with the survival time of patients with lung cancer. circUBAC2 promoted the progression of lung adenocarcinoma cells. Mechanistically, circUBAC2 competed with YAP to bind 14-3-3 proteins, thereby promoting increased nuclear entry of YAP, which activates the Hippo signaling pathway. However, the specific binding site(s) between circUBAC2 and 14-3-3 proteins remains unidentified, and the molecular mechanism by which circUBAC2 disrupts 14-3-3/YAP protein interactions remains unclear. These questions warrant further investigation.

The 14-3-3 proteins are a family of phosphoserine/threonine binding proteins, including the α/β, γ, ε, σ, ξ, θ/τ, and η subtypes (YWHAB, YWAHE, YWHAH, YWHAG, YWHAQ, YWHAZ, and stratifin [SFN]) [40]. The 14-3-3 family of proteins function through interacting with their client proteins or facilitating the interaction of other proteins, likely acting as adaptor proteins [41]. circPAK1 has been found to interact with 14-3-3zeta [42]. Herein, circUBAC2 was observed to possibly bind to 14-3-3-ε, 14-3-3-η, and 14-3-3-γ, causing them to lose their ability to bind to YAP. The 14-3-3 proteins and YAP are key factors in the Hippo signaling pathway, and 14-3-3 proteins can affect the nuclear localization of YAP [43]. As such, 14-3-3 proteins may be considered as molecular glue to exert biological effects. Small molecule stabilizers targeting the ChREBPα/14-3-3 protein–protein interaction (PPI) protect insulin-secreting β cells from glucose–lipid toxicity [44]. It is still unknown whether the interaction between 14-3-3 proteins and the YAP protein has such an effect. YAP is a multifunctional intracellular connector protein and transcriptional co-activator, playing a role in signal transduction and gene transcription regulation in normal cells [45]. After phosphorylation of YAP Ser127, a high-affinity binding site is created for 14-3-3 proteins, thereby “sequestering” YAP in the cytoplasm. This serves as the key structural basis for the Hippo pathway to inhibit the transcriptional activity of YAP [46]. YAP shares a common interaction domain with the TEAD/TEF family of transcription factors, and the entry of YAP into the nucleus enhances the activity of the TEAD/TEF family [47]. Therefore, we hypothesized that circUBAC2 activates the Hippo signaling pathway. Transcriptome sequencing suggested that circUBAC2 might also be associated with NFκB pathway. We believe that circUBAC2 might affect the progression of lung adenocarcinoma through multiple mechanisms, which are worth exploring in depth in future research.

Deubiquitinating enzymes (DUBs) are a specialized class of proteases that catalyze the removal of ubiquitin moieties (either mono- or polyubiquitin chains) from substrate proteins, thereby playing crucial regulatory roles in protein homeostasis [48]. OTUB1, a member of the OTU deubiquitinase superfamily, participates in the pathophysiological processes of various diseases including cancer, immune disorders, and renal pathologies [49, 50]. OTUB1 hydrolyzes K48- and K11-linked ubiquitin chains (with preferential activity toward K48 linkages) through its conserved OTU domain, thereby preventing proteasomal degradation of target proteins and enhancing their stability [51]. OTUB1 bound YAP through its ovarian tumor (OTU) domain and mediated deubiquitination at lysine residues K90, K280, K343, K494, and K497, thereby suppressing YAP degradation [27]. We confirmed that circUBAC2 physically interacts with OTUB1, although the precise binding domain(s) remain unidentified. circUBAC2 enhances the OTUB1–YAP interaction and potentially functions as a molecular scaffold bridging OTUB1 and YAP proteins, thereby increasing the efficiency of YAP deubiquitination. While the physical interaction between circUBAC2, OTUB1, and YAP has been observed, the structural basis underlying this complex remains elusive. Future studies should address whether this mechanism is broadly applicable.

RNA binding proteins can recognize and bind specific RNA sequences or structural elements [52]. They participate in the post transcriptional regulation of genes by regulating the translation, stability, and variable splicing of mRNAs [53]. The synthesis and splicing of circRNAs are also regulated by RBPs [29]. Herein, we observed that circUBAC2 has binding domains for DGCR8, EIF4A3, and FUS. Finally, we confirmed that the expression of circUBAC2 is regulated by EIF4A3. EIF4A3 has RNA binding domains and ATP binding domains, which can bind with transcription factors and RNA molecules to form complexes, thus participating in the entire process of transcription initiation, regulation, and termination [54]. Existing evidence suggested that a significant number of noncoding RNAs are regulated by EIF4A3. For example, circIKBKB cyclization is promoted by the direct binding of EIF4A3 to the circIKBKB flanking region, thereby acting as a pre-mRNA splicing factor [55]. Evidence suggests that EIF4A3 is involved in the regulation of long noncoding RNA CASC11 expression [56]. Our research confirmed that EIF4A3-induced circUBAC2 promoted lung adenocarcinoma progression. DGCR8 and FUS, as RBPs, can also regulate the expression of circRNAs. For example, DGCR8 maintained the stability of circKPNB1 in glioma stem cells [57]. In addition, circFndc3b interacts with the RNA binding protein FUS to regulate vascular endothelial growth factor (VEGF) expression and signaling [58]. We only briefly discuss the relationship between circUBAC2, DGCR8, and FUS in this article because it remains unclear whether circUBAC2 is regulated by DGCR8 and FUS. We speculated that the expression of circUBAC2 might be regulated by multiple factors.

**Fig. 10:**
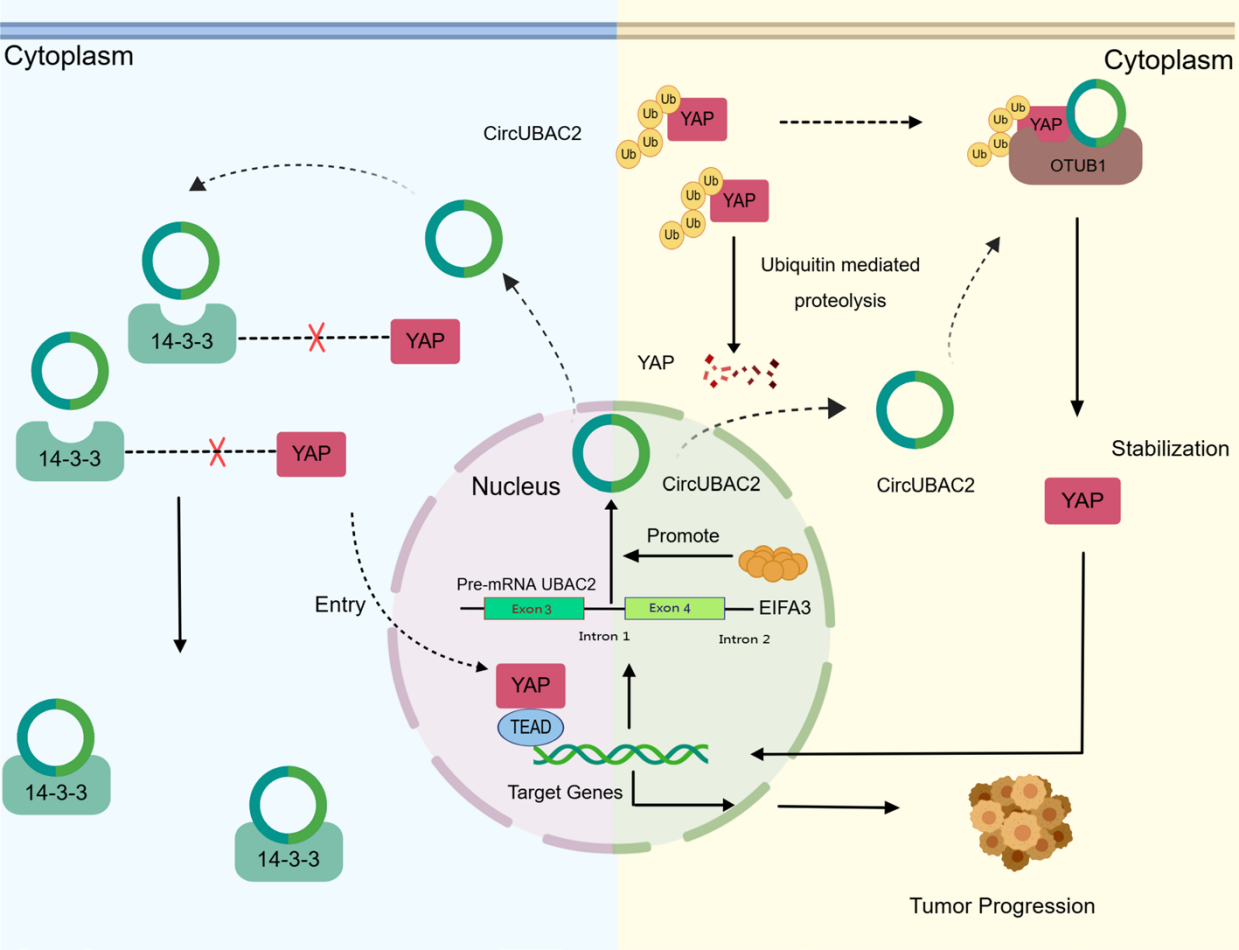
EIF4A3-induced circUBAC2 promoted lung cancer progression via regulation of the Hippo signaling pathway

In summary, the present research showed that circUBAC2 is a key factor in the progression of lung adenocarcinoma (Fig. 10).

## Conclusions

circUBAC2 is upregulated in lung adenocarcinoma tissues and correlates with poor prognosis. It drives tumor cell migration, invasion, and proliferation through dual mechanisms: as a competitive endogenous RNA, it binds 14-3-3 to disrupt YAP cytoplasmic retention, enabling YAP nuclear translocation and TEAD-mediated pro-metastatic gene activation; simultaneously, it scaffolds OTUB1–YAP interactions to enhance YAP deubiquitination and stabilization. EIF4A3 regulates circUBAC2 biogenesis via intronic binding. These findings position circUBAC2 as a promising therapeutic target for lung adenocarcinoma.

## Supplementary Information

Supplementary Material 1.

Supplementary Material 2.

Supplementary Material 3.

Supplementary Material 4.

Supplementary Material 5.

## Data Availability

No datasets were generated or analyzed during the current study.

## Abbreviations

circRNAs: Circular RNAs
NSCLC: Non-small cell lung cancer
SCLC: Small cell lung cancer
EGFR: Epidermal growth factor receptor
EIF4A3: Eukaryotic translation initiation factor 4A3
RNA-ISH: RNA in situ hybridization
qRT-PCR: Total RNA extraction and quantitative real-time PCR
MTT: 3-[4,5-Dimethylthiazol-2-yl]-2,5-diphenyltetrazolium bromide
EdU: 5-Ethynyl-2′-deoxyuridine
3D: Three-dimensional
MS: Mass spectrometry
Co-IP: Co-immunoprecipitation
ChIP: Chromatin immunoprecipitation
RBPs: RNA binding proteins
TEAD: TEA domain transcription factor
