# Supplementary material for: EIF4A3-induced circUBAC2 promotes lung cancer progression via regulation of the Hippo signaling pathway
Source: Cell Mol Biol Lett. 2026 Apr 5;31:83. doi: 10.1186/s11658-026-00912-0 (PMC13277127; doi:10.1186/s11658-026-00912-0)
Supplement: Supplementary file 1 — Supplementary Material 1. [file 11658_2026_912_MOESM1_ESM.docx]

**Figure S1**.CircUBAC2 promoted tumor growth through the YAP pathway.**A)**Western blotting detection of the levels of YAP1(nucleus) after transfection of the circUBAC2 knockdown vector(shcircUBAC2#1 and shcircUBAC2#2) and circUBAC2 overexpression vector.**B,C)** MTT assays were performed to assess the tumor growth after transfection of the circUBAC2 knockdown vector(shcircUBAC2#1 and shcircUBAC2#2) and circUBAC2 overexpression vector.


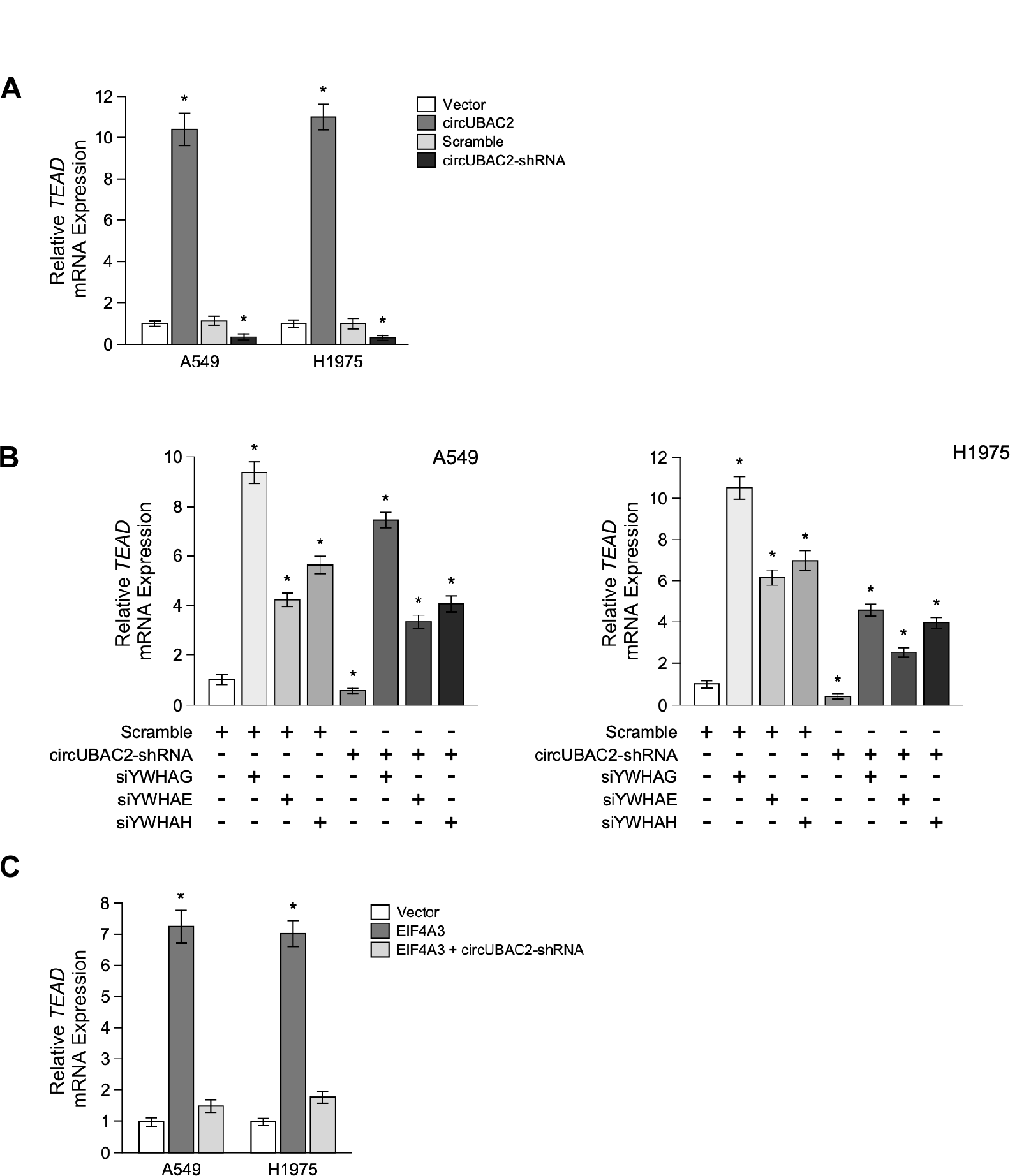


**Figure S2**.qRT-PCR assays were used to detect the expression of TEAD mRNA.**A)** qRT-PCR detection of the levels of TEAD mRNA after transfection of the circUBAC2 knockdown vector and circUBAC2 overexpression vector.**B)** qRT-PCR detection of the levels of TEAD mRNA after co-transfection of the circUBAC2 knockdown vector and YWHAG/YWHAE/YWHAH knockdown vector.A) qRT-PCR detection of the levels of TEAD mRNA after co-transfection of the circUBAC2 knockdown vector and EIF4A3 overexpression vector.





**Figure S3**.YAP1 stability decreased in circUBAC2-shRNA group compare with the scramble control group.A-C) YAP protein half-life was measured by cycloheximide chase assay in cells transfected with circUBAC2 knockdown vector and scramble vector.


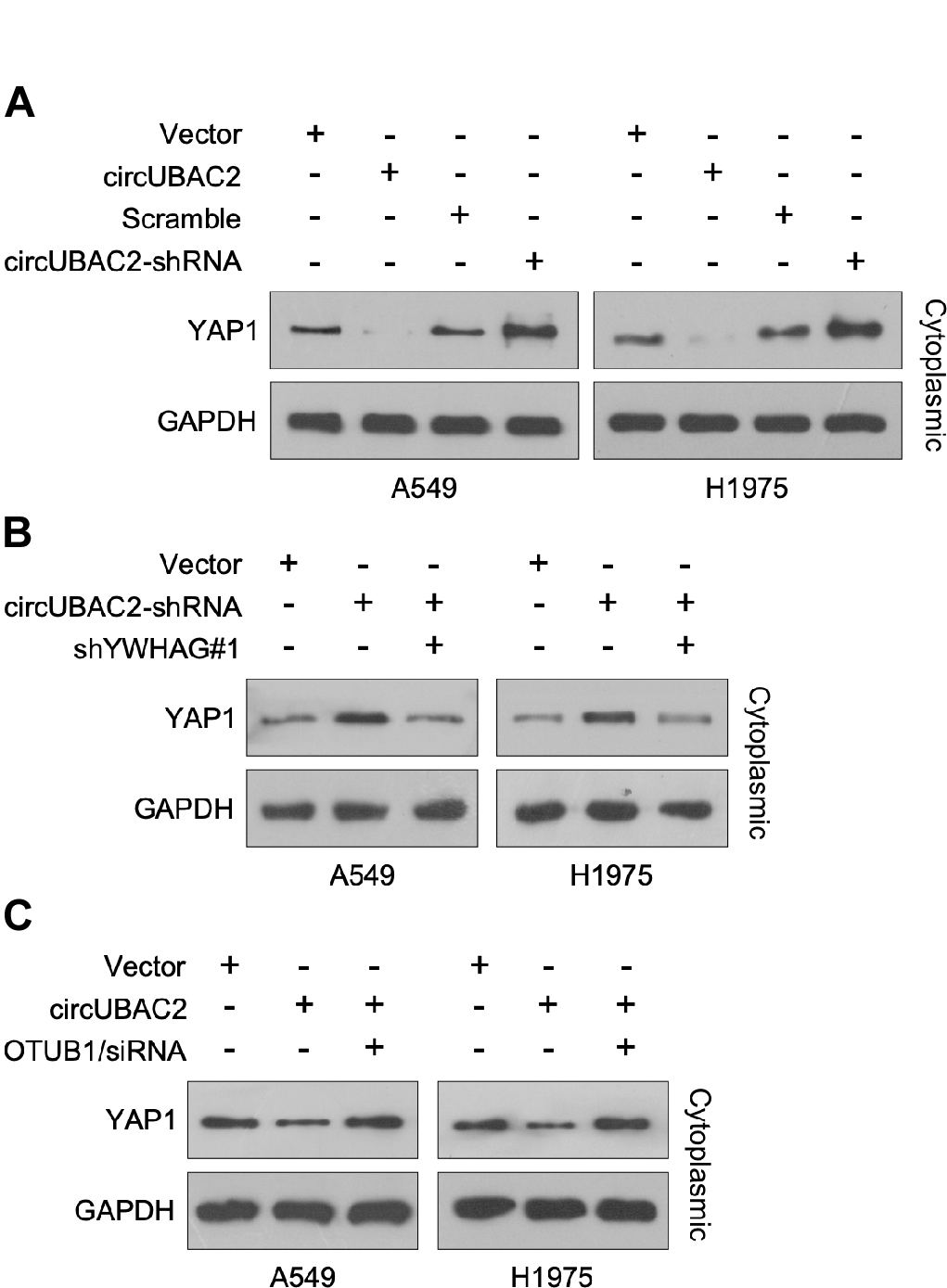


**Figure S4.**The expression level of YAP1 in the cytoplasm of esophageal squamous cell carcinoma cells.**A)** Western blotting detection of the levels of YAP1(cytoplasm) after transfection of the circUBAC2 knockdown vector and circUBAC2 overexpression vector.**B)** Western blotting detection of the levels of YAP1(cytoplasm) after co-transfection of the circUBAC2 knockdown vector and YWHAG knockdown vector. **C)** Western blotting detection of the levels of YAP1(cytoplasm) after co-transfection of the OTUB1 knockdown vector and circUBAC2 overexpression vector.


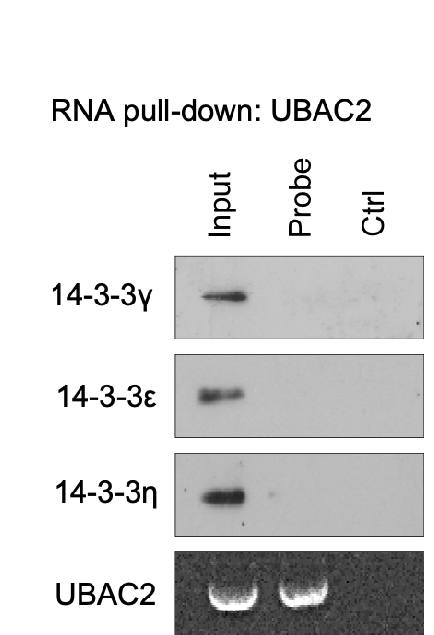


**Figure S5.**The RNA pulldown experiment detected the binding between UBAC2 mRNA and 14-3-3 protein.


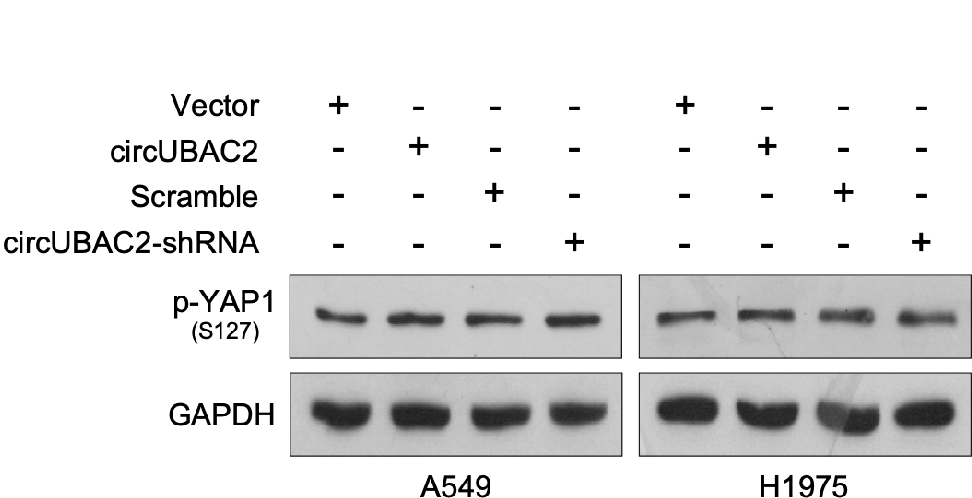


**Figure S6**.Western blotting detection of the phosphorylation level of YAP1 after co-transfection of the circUBAC2 knockdown vector and circUBAC2 overexpression vector.
