# Supplementary material for: EIF4A3-induced circUBAC2 promotes lung cancer progression via regulation of the Hippo signaling pathway
Source: Cell Mol Biol Lett. 2026 Apr 5;31:83. doi: 10.1186/s11658-026-00912-0 (PMC13277127; doi:10.1186/s11658-026-00912-0)
Supplement: Supplementary file 2 — Supplementary Material 2. [file 11658_2026_912_MOESM2_ESM.docx]

**Table S1A. Clinicopathological characteristics of studied patients in lung cancer**

|  | **Number of cases** |
| --- | --- |
| **Age(years)** |  |
| > 55 | 46 |
| ≤ 55 | 25 |
| **AJCC clinical stage** |  |
| I | 33 |
| II | 22 |
| III | 16 |
| IV | 0 |
| **T classification** |  |
| T1 | 39 |
| T2 | 23 |
| T3 | 7 |
| T4 | 2 |
| **N classification** |  |
| N0 | 43 |
| N1 | 16 |
| N2 | 10 |
| N3 | 2 |
| **M classification** |  |
| Yes | 0 |
| No | 71 |
| **Gender** |  |
| Male | 40 |
| Female | 31 |
| **Survive or Mortality** |  |
| Survive | 23 |
| Mortality | 48 |
| **Expression of *circUBAC2*** |  |
| High | 45 |
| Low | 26 |

**Table S1B. The expression of *circUBAC2* in lung cancer**

| **Expression of *circUBAC2*** |  |
| --- | --- |
| Negative | 0 (0%) |
| Positive | 71 (100%) |
| Low expression | 26 (36.7%) |
| High expression | 45 (63.3%) |
