# Supplementary material for: EIF4A3-induced circUBAC2 promotes lung cancer progression via regulation of the Hippo signaling pathway
Source: Cell Mol Biol Lett. 2026 Apr 5;31:83. doi: 10.1186/s11658-026-00912-0 (PMC13277127; doi:10.1186/s11658-026-00912-0)
Supplement: Supplementary file 3 — Supplementary Material 3. [file 11658_2026_912_MOESM3_ESM.docx]

Table S2A. Primers for RT-qPCR

| Primers | Sequences (5’-3’*) |  |
| --- | --- | --- |
| CCN2: | F: CAGCATGGACGTTCGTCTG  R: AACCACGGTTTGGTCCTTGG |  |
| SNAI2：  axin2 :  SOX2:  CCN1:  circUBAC2:  OTUB1  YWHAG:  YWHAE :  YWHAH:  DGCR8:  EIF4A3:  FUS:  β-actin:  GAPDH  UBAC2：  circUBAC2: | F: CGAACTGGACACACATACAGTG  R:CTGAGGATCTCTGGTTGTGGT  F: CAACACCAGGCGGAACGAA  R: GCCCAATAAGGAGTGTAAGGACT  F: TGGACAGTTACGCGCACAT  R:CGAGTAGGACATGCTGTAGGT  F: ACCGCTCTGAAGGGGATCT  R: ACTGATGTTTACAGTTGGGCTG  F:CCAAGAGTCCAAGTGGCACA  R:CACAGGTGCCAGGACTAGT  F:TCGGTCCTATACAAGGAGTATGC  R：GGTCTTGCGGATGTACGAGT  F:AGCCACTGTCGAATGAGGAAC  R:CTGCTCAATGCTACTGATGACC  F:GATTCGGGAATATCGGCAAATGG  R:GCTGGAATGAGGTGTTTGTCC  F:TGGCTGATGGAAACGAAAAGAA  R:CCTCTGCTAAGTAGCGGTAGT  F:GCAGAGGTAATGGACGTTGG  R:AGAGAAGCTCCGTAGAAGTTGAA  F:GGGGCATCTACGCTTACGG  R:GCGATGACATCTCTCCCTTTGA  F:ATGGCCTCAAACGATTATACCCA  R:GTAACTCTGCTGTCCGTAGGG  F:TGGATCAGCAAGCAGGAGTA  R:TCGGCCACATTGTGAACTTT  F:GGAGCGAGATCCCTCCAAAAT  R:GGCTGTTGTCATACTTCTCATGG  F:GCTCCAGTGGGCTCTACAAG  R:AAGAGCTTCTGGCAGTGAGG  F:CCAAGAGTCCAAGTGGCACA  R:CACAGGTGCCAGGACTAGT |  |
