# Supplementary material for: EIF4A3-induced circUBAC2 promotes lung cancer progression via regulation of the Hippo signaling pathway
Source: Cell Mol Biol Lett. 2026 Apr 5;31:83. doi: 10.1186/s11658-026-00912-0 (PMC13277127; doi:10.1186/s11658-026-00912-0)
Supplement: Supplementary file 4 — Supplementary Material 4. [file 11658_2026_912_MOESM4_ESM.docx]

Table S3A. Target sequences of shRNAs and siRNA

| Targets | Sequences (5’-3’*) |
| --- | --- |
| circUBAC2 shRNA target sequence-1 | GTAGCCATAAGTGGACTTCCT |
| circUBAC2 shRNA target sequence  -2 | GCCATAAGTGGACTTCCTGGC |
| circUBAC2 shRNA target sequence-3 | GGACTTCCTGGCACCTGTGTT |
| YWHAG: | GAACGTGACAGAGCTGAATGA |
| YWHAE: | GCGCCTCCATCCTTTATACTT |
| YWHAH: | GCTTGACAAGTTCCTGATCAA |
| DGCR8: | GCTCGATGAGTTAGAAGATTT |
| EIF4A3: | AACGAGCAATCAAGCAGATCA |
| FUS: | GGTGGAGGTGGAGGTAACTAT |
| siOTUB1#1 | CCTATACAAGGAGTATGCTGAAGAT |
| siOTUB1#2 | GAGTATGCTGAAGATGACAACATCT |
| siOTUB1#3 | GGACACTACGATATCCTCTACAAAT |

Table S3B.Various RNA probe sequences

| Targets | Sequences (5’-3’*) |
| --- | --- |
| circUBAC2(Fish/ISH probe ) : | ACAGGTGCCAGGAAGTCCACTTATG |
