# Supplementary material for: EIF4A3-induced circUBAC2 promotes lung cancer progression via regulation of the Hippo signaling pathway
Source: Cell Mol Biol Lett. 2026 Apr 5;31:83. doi: 10.1186/s11658-026-00912-0 (PMC13277127; doi:10.1186/s11658-026-00912-0)
Supplement: Supplementary file 5 — Supplementary Material 5. [file 11658_2026_912_MOESM5_ESM.docx]

Table S4. Antibody concentration

| Antibody | concentration |  |
| --- | --- | --- |
| DGCR8(Abcam,Cambridge, MA):  EIF4A3(Abcam,Cambridge, MA):  FUS(Abcam,Cambridge, MA): | 1:1000  1:1000  1:1000 |  |
| p21(Abcam,Cambridge, MA)：  MMP2(Abcam,Cambridge, MA):  MMP9(Abcam,Cambridge, MA):  H3-ChIP(Abcam,Cambridge, MA):  YAP1(Abcam,Cambridge, MA):  YAP1-IF(Abcam,Cambridge, MA):  YWHAE(Cell Signaling, Danvers, MA):  YWHAG(Cell Signaling, Danvers, MA):  YWHAH(Cell Signaling, Danvers, MA):  H3(Abcam,Cambridge,MA):  GAPDH(Abcam,Cambridge, MA):  OTUB1(Affinity Biosciences，Melbourne，Australia )  Ubiquitin(aladdin,Shanghai,China) | 1:1000  1:1000  1:2000  1:1000  1:1000  1：250  1:1000  1:1000  1:1000  1:1000  1:2000  1:1000  1:1000 |  |
